# FPR1 affects acute rejection in kidney transplantation by regulating iron metabolism in neutrophils

**DOI:** 10.1186/s10020-025-01077-w

**Published:** 2025-01-23

**Authors:** Peiyuan Li, Wenbin Ji, Baotong Zhang, Haowen Jia, Jinmiao Wang, Zhaonan Sun, Yifan Wang, Weiwei Wang, Feng Qi

**Affiliations:** 1 Department of General Surgery, Tianjin Medical University General Hospital, No. 154, Anshan Road, Heping District, Tianjin, 300052 China; 2 Department of General Surgery, Tianjin Medical University General Hospital Airport Hospital, No.85, East Sixth Road, Dongli District, Tianjin, 300300 China; 3 Department of Breast and Thyroid Surgery, Tianjin Union Medical Center, Nankai University, Tianjin, 300121 China; 4 Department of General Surgery, Tianjin Baodi Hospital, Tianjin Medical University Baodi Hospital, #8 Guangchuan Road, Baodi, 301800 Tianjin, China

**Keywords:** Acute rejection, Kidney transplantation, FPR1, Neutrophil, Iron metabolism

## Abstract

**Background:**

Acute rejection (AR) is one of the significant factors contributing to poor prognosis in patients following kidney transplantation. Neutrophils are the main cause of early host-induced tissue injury. This paper intends to investigate the possible mechanisms of neutrophil involvement in acute rejection in renal transplantation.

**Methods:**

Samples were analyzed for their relationship with immune cells using CIBERSORT. WGCNA was used to identify modules with high relevance to neutrophils and hub genes in the modules were extracted. The effect on neutrophil function after blocking formyl peptide receptor 1 (FPR1) was tested in vitro experiments. The effects of blocking FPR1 on neutrophil function as well as acute rejection were tested in vivo after constructing a mouse kidney transplant model.

**Results:**

The proportion of neutrophils was higher in the AR group than in the non-rejection group, and FPR1 was identified as an important gene in the regulation of acute rejection in kidney transplantation by neutrophils. At the cellular level, blocking FPR1 inhibited the activation of the ERK1/2 pathway, decreased ferrous ion content, affected the expression of iron metabolism-related proteins, and suppressed the formation of NETs. In the acute rejection model of renal transplantation, blockade of FPR1 decreased graft neutrophil infiltration and NETs content. Meanwhile, blocking FPR1 attenuated graft injury during acute rejection.

**Conclusion:**

This study found that FPR1 might be an important molecule involved in neutrophils during acute rejection of kidney transplantation, explored the relationship between kidney transplantation and neutrophils, and provided potential treatment methods for clinical practice.

**Supplementary Information:**

The online version contains supplementary material available at 10.1186/s10020-025-01077-w.

## Introduction

Kidney transplantation is the first choice for patients with end-stage renal failure. The survival rate and quality of life of patients after transplantation are better than those after continuous dialysis (Wolfe et al. 1999; Purnell et al. 2013). Although current immunosuppressive regimens have prolonged the survival rate and time of patients and grafts, immune tolerance is rare. In addition, acute rejection (AR) and subclinical rejection events can limit graft and patient prognosis after renal transplantation (Schwarz et al. 2005; Zhao et al. 2014).

Neutrophils are the principal cause of early host-induced tissue injury due to their capacity to coordinate the subsequent influx of white blood cells into the graft. Neutrophils produce neutrophil extracellular traps (NETs) that kill bacteria outside the cell (Brinkmann et al. 2004). NETs can be produced in sterile inflammation and play different roles in atherosclerosis (Warnatsch et al. 2015), vasculitis (Kessenbrock et al. 2009), and autoimmune diseases (Hakkim et al. 2010). A study shown that the formation of NETs was enhanced in the patients with rejection after kidney transplantation (Torres-Ruiz et al. 2020). Meanwhile, neutrophils produce reactive oxygen species (ROS), which can cause further tissue damage (Grommes and Soehnlein 2011). Limiting the initial penetration of neutrophils has been shown to minimize the need for the recruitment of other inflammatory cells and resulting tissue injury (Awad et al. 2009). Neutrophils are the most abundant circulating leukocytes and the cell population that has an early response to infiltrating cells and solid organ xenografts after transplantation. Experimental strategies to remove neutrophils or inhibit their early penetration have been successful in mitigating acute rejection and enhancing graft function and survival (al-Mohanna et al. 1996, 1997; Sachs et al. 2007; Honda et al. 2013; Shimizu et al. 2012).

Gene microarrays have been widely used in transplantation immunology over the past decade (Stegall et al. 2002). The data from renal biopsy and liquid biopsy provide potential molecular characteristics and enable accurate assessment of the immune status of allograft recipients (Lin and Lin 2017). Because neutrophils play an important role in transplantation and to better understand the changes and underlying molecular regulation of immune status in transplant recipients, this study recruited the relevant dataset GSE14346 after kidney transplantation from the Gene Expression Omnibus (GEO) database. By studying the relationship between neutrophil-related genes and acute rejection in kidney transplantation, we found that formyl peptide receptor 1 (FPR1) may play an important role.

Neutrophils rely on cell surface receptors in G-protein-coupled receptor (GPCR) family to sense inflammatory stimuli and their surroundings. Through our analysis we identified FPR1 as a possible key gene for neutrophil function during acute rejection of kidney transplantation. FPR1 was the first GPCR identified in human neutrophils. Activation of FPR1 leads to a cascade of events in the neutrophil, involving phagocytosis, degranulation, ROS generation, and chemotaxis (Boulay et al. 1990; Ye et al. 2009). When FPR1 is blocked, superoxide production, elastase master, and chemotaxis of neutrophils activated by N-Formyl-Met-Leu-Phe (fMLF) are reduced (Yang et al. 2013; Mills et al. 2000).

Studies on FPR1 and neutrophils in acute rejection of renal transplantation remain scarce. Therefore, our aim was to investigate whether FPR1 could influence renal transplantation acute rejection and its possible mechanisms by affecting neutrophil function and ultimately provide new insights into the involvement of neutrophils in renal transplantation acute rejection.

## Materials and methods

### Data download and processing

We used the GEO database for transplantation retrieval. Neutrophils in peripheral blood interact with damage-associated molecular patterns (DAMPs) through pattern recognition receptors, thereby exacerbating graft damage (Braza et al. 2016; McDonald et al. 2010). Based on the role of neutrophils in transplant immunity, we selected the GSE14346 dataset from the GEO database for our study. GSE14346 (Li et al. 2012) consists of peripheral blood samples from kidney transplantation, which include samples exhibiting acute rejection as well as those without rejection (Named STA in the database). There were 38 samples in the AR group and 37 samples in the STA group. Differentially expressed genes (DEGs) in dataset microarrays were screened using the limma package in R software (Ritchie et al. 2015). The cutoff conditions were set to an adjusted P value < 0.05 and an absolute value of log-fold change| log2FC| > 0.8.

GEO2R is an interactive online software application that allows users to compare two or more groups of samples in a certain platform in order to identify genes that are differentially expressed across different subtypes or different diseases. The datasets (GSE131179 and GSE142667) were used to identify that FPR1 and CSF3R were high expressed in acute rejection group by GEO2R (Verma et al. 2020).

We used the public dataset of scRNA-seq from the NCBI BioProject (accession number PRJNA974568) (Shi et al. 2023). Sequencing data were downloaded using Aspera, aligned to the human reference genome (GRCh38), and processed using the Cell Ranger 8.0.0 count pipeline (10x Genomics) to obtain the gene expression matrix. The raw gene expression matrix was processed to remove doublets using the DoubletFinder R package (McGinnis et al. 2019). Cells were filtered using the Seurat R package (Hao et al. 2024) based on the following criteria: cells with > 200 genes and < 20% mitochondrial gene expression in UMI counts. The SCTransform (Lause et al. 2021) normalization method was applied with the top 3000 highly variable genes. Batch effect was removed by harmony R package. Thirty principal components were selected for cell clustering and UMAP visualization. Cell major populations were defined according to known cell-specific markers. For macrophages, the corresponding cell subsets were extracted for reanalysis, including data normalization and cell clustering.

### Functional enrichment analysis of DEGs

The ggplot2 package in R was used to generate a volcano map for differential gene analysis. Gene Ontology (GO) and Kyoto Encyclopedia of Genes and Genomes (KEGG) enrichment analyses were performed using the clusterProfiler package in R. The org.Hs.eg. db package in R was used for ID conversion. The ClusterProfiler package in R software was used for Gene Set Enrichment Analysis (GSEA) (Subramanian et al. 2005), and the gene set database was MSigDB Collections. Visualization of GSEA was performed using the ggplot2 package in R software.

### Evaluation of the relationship between immune cells and samples

CIBERSORT is a method for calculating immune infiltration (Newman et al. 2019). CIBERSORT uses linear support vector regression to mix data deconvolution (Newman et al. 2015). CIBERSORT was used to assess the status of immune cells in the samples. The sample dataset was used as the input of gene expression, and LM22 (22 immune cell types) was set as the signature gene file. A follow-up analysis of the immune cells was then performed.

### Screening of neutrophil-related genes

Weighted Gene Co-Expression Network Analysis (WGCNA) is a systems biology method used to describe gene association patterns between different samples. It uses gene information to identify gene sets of interest and conduct significant association analysis with phenotypes (Langfelder and Horvath 2008). We used the presentation data retrieved from GEO for WGCNA. The R package “WGCNA” was applied to search for modules and central genes associated with immune cells. The adjacency matrix was transformed into a topological overlap matrix (TOM). Genes were divided into different gene modules based on TOM-based dissimilarity measures. We clustered the samples, selected cutHeight = 200 to filter the cluster tree, and set the minimum number of samples for each cluster to 10. Here, we set the soft threshold power to 14 to identify critical modules. The module with the highest correlation with immune cells was selected for hub gene screening. Module membership indicates the correlation between genes and modules, while Gene Significance reflects the correlation between genes and phenotypic traits. These two parameters are utilized for the identification of key genes. Hub genes were defined as having gene significance > 0.3 and module membership > 0.8.

Protein–protein interaction (PPI) network analysis was performed on the screened module genes using the STRING website (Szklarczyk et al. 2015). CytoHubba in Cytoscape was used to screen hub nodes (Shannon et al. 2003), and the top three genes were selected as hub genes for further analysis.

### Assessing the diagnostic efficacy of hub genes

ROC analysis by R was performed to predict the diagnostic validity of biomarkers. The area under the ROC curve (AUC) value was used to determine the diagnostic validity of distinguishing rejected and nonrejected samples after kidney transplantation in the GSE14346 dataset.

### Mouse kidney transplantation

Intraperitoneal heterotopic kidney transplantation was conducted, as mentioned previously (Wang et al. 2017). Briefly, the donor’s kidneys were taken from BALB/c or C57BL/6 mice and transplanted into C57BL/6 recipients’ abdominal cavities by microsurgical techniques. Kidney transplantation from C57BL/6 to C57BL/6 mice was considered a syngeneic group. Kidney transplantation from BALB/c to C57BL/6 mice was considered the allogeneic group. fMLF is an endogenous chemotactic peptide and serves as an agonist for FPR1^22^. HCH6-1 is a potent, competitive dipeptide antagonist of FPR1. HCH6-1 inhibits the specific activation of neutrophils by fMLF (Yang et al. 2017). Therefore, the allogeneic group was treated with HCH6-1 and was considered the allogeneic + HCH6-1 group. HCH6-1 (50 mg/kg) was intraperitoneally injected into mice one day before surgery and one hour after surgery. On the seventh day after the operation, the mice were euthanized for follow-up experiments.

### Extraction and grouping of neutrophils from mouse bone marrow

Neutrophils were extracted from mouse bone marrow according to the previous scheme (Boxio et al. 2004). Following mice euthanization, the femurs and tibia were extracted. Cells in bone marrow were filtered with a 70 µM cell filter and centrifuged (600 x g, 4 °C, 5 min), then re-suspended in 3mL neutrophil separation buffer. The cell solution was positioned on an intermittent Percoll gradient, and the 1500xg was rotated for 30 min at 4 °C. Neutrophils were absorbed between 78% and 69% of the percoll density. Neutrophils were collected with a pipette and washed with a neutrophil washing solution. Neutrophils were blocked for Fc receptors (anti-CD16/32 antibody, BioLegend, USA) and then stained with (Zombie dye, APC-Cy7, BioLegend, USA) and anti-Ly6G antibody (FITC, BioLegend, USA). The purity of neutrophils was above 85% as determined by flow cytometry. To simulate the inflammatory environment in the body, neutrophils were divided into two groups: (1) Control group (Con): neutrophils without treatment. (2) Stimulation group (Sti): neutrophils pretreated with LPS (1 µg/mL) for 1 h.

HCH6-1(MCE, China) is an effective competitive dipeptide antagonist of FPR1. To verify the role of HCH6-1 in the inflammatory environment, neutrophils were divided into three groups: (1) LPS group (LPS): Neutrophils from bone marrow were supplemented with LPS (1 µg/mL) for 1 h. (2) LPS + fMLF group (LPS + fMLF): LPS-activated neutrophils from bone marrow (1 µg/mL) for 1 h, and then treated with fMLF (30nM) for 12 h. (3) LPS + fMLF + HCH6-1 group (LPS + fMLF + HCH6-1): Neutrophils were pretreated with HCH6-1 (3µM) for 15 min, and then the operation was the same as in LPS + fMLF group.

### Real-time polymerase chain reaction (RT-PCR)

RT‒PCR was utilized to identify gene expressions mentioned in this article. The specimens and cell lines were fully lysed utilizing TRIzol (Invitrogen, CA, USA), and total RNA was extracted. Utilizing a cDNA reverse transcription kit (Vazyme, Nanjing, China), isolated RNA was reverse-transcribed into cDNA. Genes associated with expression in this study were analyzed using the Real Universal Color PreMix (SYBR Green) RT-PCR kit (Vazyme, Nanjing, China). The mRNA primers were designed by Keyybio (Jinan, China).

The relative expression of genes was analyzed using the 2^-ΔΔCt^ method and was replicated three times for each group and analyzed using T-test or ANOVA. The primer sequences are as follows:

GAPDH (Mus)

Forward: GGTGAAGGTCGGTGTGAACG

Reverse: CTCGCTCCTGGAAGATGGTG

FPR1 (Mus).

Forward: CCCTGGACCAAAGATCCTGT

Reverse: ATGGACATGGGAGTGCTGAA

CSF3R (Mus)

Forward: GTTACCCAGCTTCTGGGACT

Reverse: TTGGAGCACATAGGCCTGAA

Ftl (Mus).

Forward: CTTCCAGGATGTGCAGAAGC

Reverse: CAGATCCAAGAGGGCCTGAT

Fth (Mus)

Forward: TTGACCGAGATGATGTGGCT

Reverse: CAGTGCACACTCCATTGCAT

Tfrc (Mus).

Forward: ACCTGGGCTATTGTAAGCGT

Reverse: CTCAGCTGCTTGATGGTGTC

Ferroportin (Mus)

Forward: CCACTCTCTCTCCACTTGGG

Reverse: AGCTGTCAAGAGAAGGCTGT

### H&E staining

Kidney samples were preserved with 10% formalin for 48 h. Fixated tissues were encapsulated in paraffin wax and prepared into 5 μm sections for H&E staining. The Paller score, which was used to evaluate the renal tubules, was then used to blindly measure the degree of kidney injury (Zhang et al. 2022). The specific scoring criteria are summarized as follows: A total of 100 cortical tubules from at least 10 different regions of each kidney are evaluated, with higher scores indicating more severe damage. The assessment includes the presence and extent of tubular epithelial cell flattening, loss of brush border, formation of membrane vacuoles, interstitial edema, cytoplasmic vacuolization, cell necrosis, and tubular lumen obstruction.

### Enzyme-linked immunosorbent assay (ELISA)

To fully assess the inflammatory environment in each group, serum was collected and measured using ELISA kits (DAKEWE, Shenzhen, China) for TGF-β1, TNF-α, and IL-6 levels. All the above indicators are tested per the manufacturer’s guidelines.

### Detection of nets formation in vitro

To assess neutrophil Nets formation, cell supernatant and serum were collected, and NE and MPO levels were assessed using ELISA kits (CLOUD-CLONE, Wuhan, China). Besides, we employed Zombie NIR (an amine-reactive dye, APC-Cy7, Biolegend, USA) to exclude necrotic cells and then a DNA dye (7-AAD, Percp-cy5.5, Yeasen Biotechnology, Shanghai, China) to detect extracellular DNA as a symbol of Nets formation by using flow cytometry [20, 21].

### Immunohistochemistry staining

Following dewaxing, rehydration, antigen repair, and endogenous peroxidase inactivation, the prepared kidney tissue sections were incubated overnight at 4℃ with primary antibodies: anti-Ly6 G (Servicebio, Wuhan, China), FPR1 (Bioss, Beijing, China), CSF3R (Bioss, Beijing, China), NE (Servicebio, Wuhan, China), MPO (Servicebio, Wuhan, China) monoclonal antibody. The second day was then washed, and the secondary antibody (Santa Cruz Biotechnology, USA) was applied. Following sections were rewashed in phosphate buffer; they were incubated with a streptavidin-biotin peroxidase complex at 37℃ for 30 min. Slices were reacted with diaminobenzidine (Sigma, USA) as a chromogenic agent and then stained with hematoxylin. Specific brown staining observed in immunohistochemical staining experiments under a light microscope was considered as positive cells. Under a light microscope, the numbers of FPR1-, CSF3R-, MPO-, NE- and LY6G- positive cells in 5 diferent felds in each section were counted by two different pathologists, and finally the individual samples were analyzed with the mean of the positive cell counts.

### Immunofluorescence staining

To clarify ROS levels in the kidneys of each group, Kidney tissue was collected and made into frozen slices. The histochemical pen drew a circle around the tissue. The autofluorescence quenching agent was added for 5 min. The tissue was then rinsed with running water. The ROS probes (Dihydroethidium, BestBio, Nanjing, China) were dripped into the circle. After that, it was incubated in an incubator for 30 min. The glass slides were rinsed with PBS three times, each time 5 min. DAPI dye solution was applied and incubated at room temperature (RT) without light for 10 min. They were observed and photographed under a fluorescence microscope. The fluorescence intensity of ROS in each group of grafts was calculated by using image J and used for subsequent analysis.

### Western blotting

Pretreated neutrophils were fully lysed in RIPA lysis buffers containing protease inhibitors(phenyl methane sulfonyl fluoride, Servicebio, Wuhan, China). Before heating denaturation, protein solution sticentration was determined utilizing a BCA kit (Yeasen Biotechnology, Shanghai, China) and adjusted to the same concentration. Each group’s total protein was separated utilizing SDS-PAGE and then translocated to a PVDF membrane. PVDF membranes were blocked with 5% skimmed milk before being incubated with the following primary antibodies: anti-ERK1/2 (1:1000, Affinity Biosciences); Anti-Phospho-ERK1/2 (1:1000, Affinity Biosciences); anti-Ferritin Light Chain (1:1000, Abclonal); anti-Ferroportin 1 (1:1000, Affinity Biosciences); anti-Anti Ferritin Heavy Chain (1:1000, Abclonal) and anti-Transferrin receptor protein 1 (1:1000, Affinity Biosciences) at 4℃ overnight. On the second day, a secondary antibody (anti-rabbit, 1:1000, Affinity Biosciences) was treated on PVDF membranes for 30 min at RT. The strips were soaked with ECL solution and exposed to preserve the photos.

Protein expression levels of FPR1(anti-FPR1 antibody, 1:1000, Affinity Biosciences), Ly6G(anti- Ly6G antibody,1:1000, Affinity Biosciences) and CSF3R(anti-CSF3R antibody,1:1000, Affinity Biosciences) were examined in kidney grafts according to the above procedure.

Image J software was used to calculate and analyze the gray value of the acquired images.

### Flow cytometry analysis

Splenic T cells are an important reference index for observing immune rejection in kidney transplantation (Zimmerer et al. 2022; Rosales et al. 2022; Zhang et al. 2021). Spleen samples were collected from each group of mice and cell suspensions were prepared for flow staining. Total T cells were labeled with fluorescent antibodies anti-CD3 (FITC), anti-CD4 (APC), and anti-CD8 (PerCP-Cyanine 5.5). All cell staining procedures were as per the manufacturer’s guidelines. The fluorescent antibody was purchased from BioLegend (USA). FlowJo software was used to analyze the results.

### Determination of ROS generation

ROS produced in neutrophils was detected by ROS detection kit (Beyotime, Shanghai, China) according to the manufacturer’s protocol. Each group’s fluorescence intensity was measured by a fluorescence enzyme labeling instrument.

### Chemotaxis assay

A 24-well microchemotactic chamber was used to measure cell migration (Corning, USA). After LPS stimulation, neutrophils were suspended at an overall concentration of 5 × 10^6^ cells /ml. The LPS group was supplemented with LPS only. The LPS + fMLF group was treated with LPS, and fMLF (30nM) was added to the lower chamber. The LPS + fMLF + HCH6-1 group was pretreated with HCH6-1 for 15 min in advance, and the treatment was the same as LPS + fMLF group. After incubation at 37 ℃ for 8 h, five random fields were selected, and migrated neutrophils number was counted by microscope (Olympus, Japan).

### Detection of ferrous ions in neutrophils

Iron, as an essential transition metal element in biological systems, participates in various physiological activities. Free iron typically exists in its stable redox states, namely ferrous ions and ferric ions. FerroOrange (Dojindo, Japan), as a fluorescent probe for the detection of ferrous ions, enables the assessment of ferrous ion levels in living cells.The methodology is summarized as follows:

Add DMSO to the tube containing FerroOrange, and vortex to mix thoroughly, resulting in a 1 mmol/L solution of FerroOrange. Dilute the 1 mmol/L FerroOrange solution with serum-free culture medium to prepare the desired concentration. Neutrophils were extracted from mice and subjected to corresponding treatments for each group. A solution of FerroOrange at a concentration of 1 micromolar/L was added, and the samples were incubated in a cell culture incubator. The fluorescence intensity of neutrophils in each group was measured using a fluorescence microplate reader (emission wavelength: 543 nm; excitation wavelength: 580 nm).

### Experimental animals and ethics statement

In this study, 20–25 g healthy adult male BALB/C mice and C57BL/6 J were used. Mice had unrestricted access to food and water and were acclimatized for one week. The Tianjin Medical University General Hospital Animal Ethical and Welfare Committee accepted the above animal usage protocol (IRB2023-DW-08).

### Statistical analysis

The data are represented as the mean ± standard deviation. The statistical analyses were done with R (Version 3.6.3). Group comparisons were made utilizing a one-way analysis of variance. A p-value < 0.05 is regarded as statistically significant.

## Results

### The acquisition of DEGs and comparison of relevant immune cells in the STA and AR groups

**Fig. 1:**
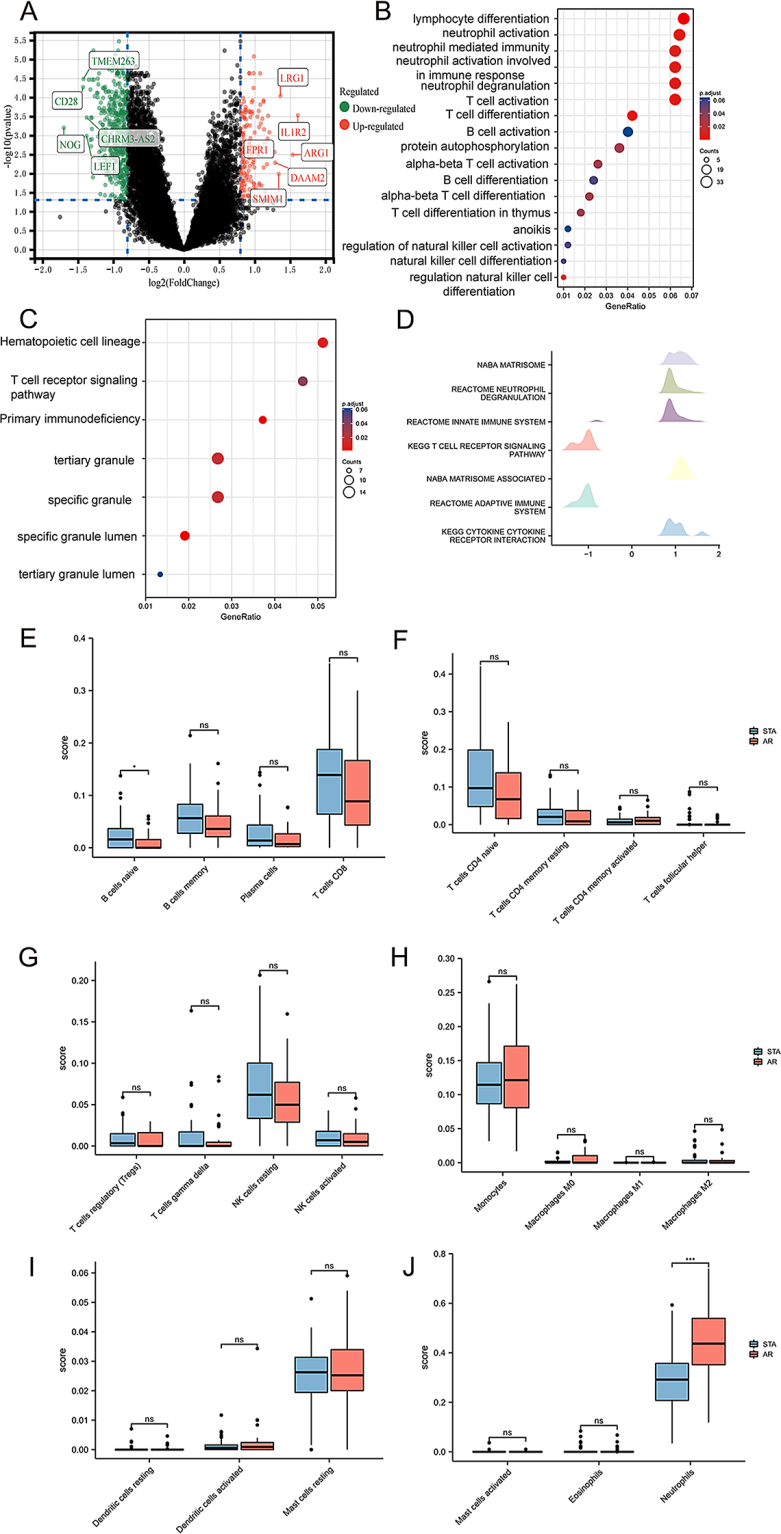
Screening and analysis of DEGs in the kidney transplant dataset and immunocorrelation analysis of the dataset samples. (**A**) Volcano plot of differentially expressed genes in GSE14346. (**B**–**C**) GO and KEGG analyses of the common DEGs. (**D**) GSEA of DEGs. (**E**–**J**) Comparison of immune cells in the STA group and the AR group. DEGs, differentially expressed genes; GO, Gene Ontology; KEGG, Kyoto Encyclopedia of Genes and Genomes; GSEA, Gene Set Enrichment Analysis; *, *p* < 0.05; **, *p* < 0.01; ***, *p* < 0.001

GSE14346 was analyzed with R and screened for DEGs. In this study, 38 AR samples and 37 STA samples from the dataset were involved. A total of 155 upregulated and 445 downregulated genes were filtered from GSE14346. The DEGs were displayed by a volcanic plot, and ggplot2 was used for visualization (Fig. 1A).

Functional enrichment analysis of 600 differentially expressed genes was performed by GO and KEGG (Fig. 1B–C). DEGs were strongly correlated with lymphocyte differentiation, neutrophil activation and neutrophil-mediated immunity (Fig. 1B). In GSEA,

REACTOME_NEUTROPHIL_DEGRANULATION and.

REACTOME_INNATE_IMMUNE_SYSTEM were activated in the AR group. KEGG_T_CELL_RECEPTOR_SIGNALING_PATHWAY and.

REACTOME_ADAPTIVE_IMMUNE_SYSTEM were weakly inhibited in the AR group (Fig. 1D).

Then we divided the GSE14346 dataset into STA and AR groups, and analyzed the relationship of 22 immune cells in different subgroups by CIBERSORT. We compared the immune cells of the STA group and AR group and found that the proportion of naive B cells in the STA group was higher than that in the AR group (Fig. 1E), and the proportion of neutrophils in the STA group was lower than that in the AR group (Fig. 1J). And the results were statistically significant (*P* < 0.05) (Fig. 1E–J).

### Extraction of neutrophil-related modules in the AR group and analysis of hub genes and immune cells

**Fig. 2:**
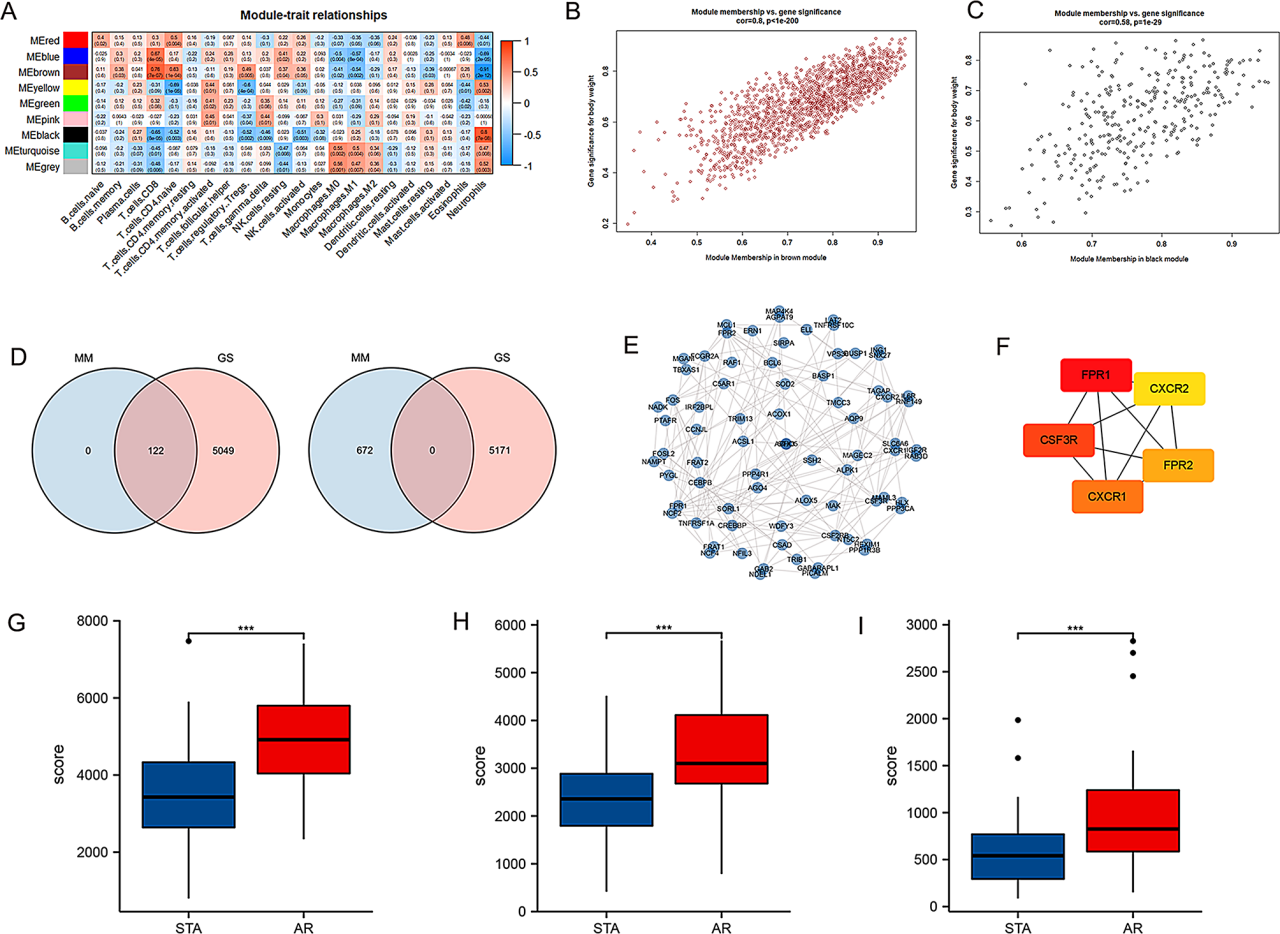
Key neutrophil-related genes identified through WGCNA and PPI networks. (**A**) Heatmap of the correlation of module signature genes with immune cell signatures. Each cell contains the correlation coefficient and P value. (**B**) A scatter plot of the brown module in neutrophils. (**C**) A scatter plot of the black module in neutrophils. (**D**) Screening and extraction of black module genes and brown module genes in neutrophils; MM: module membership; GS: Gene Significance. (**E**) PPI analysis of module genes. (**F**) Screening hub genes by CytoHubba. (**G**) Expression level of FPR1 in the STA and AR groups. (**H**) Expression level of CSF3R in the STA and AR groups. (**I**) Expression level of CXCR1 in the STA and AR groups. PPI, Protein–protein interaction; *, *p* < 0.05; **, *p* < 0.01; ***, *p* < 0.001

We performed WGCNA on the dataset to screen for modules closely related to immune cells (Supplementary Fig. 1 and Fig. 2A). Generating a heatmap of the correlation between modules and immune cells, we found that the brown and black modules of neutrophils had higher correlations and smaller P values (*P* < 0.001) (Fig. 2B-C). The Gleason score of the brown module for neutrophils had a correlation coefficient = 0.8, with *P* < 1e-200 (Fig. 2B). The Gleason score of the black module for neutrophils had a correlation coefficient = 0.58, with *P* < 1e-29 (Fig. 2C).

First, we screened and extracted the genes of the three hub modules according to module membership > 0.8 and gene significance > 0.3 (Fig. 2D). PPI network analysis was performed on the module genes, and CytoHubba was used to screen the first three hub genes (Fig. 2E–F). FPR1, CSF3R, and CXCR1 were screened out. The expression levels of FPR1 (Fig. 2G), CSF3R (Fig. 2H), and CXCR1 (Fig. 2I) in the AR group were statisticaly higher than those in the STA group (*P* < 0.05). Consistent with the single-cell data, the expression levels of FPR1 and CSF3R were found to be reduced in patients without rejection (Verma et al. 2020) (Supplementary Fig. 2).

### Expression of hub genes in the single-cell dataset of kidney transplantation acute rejection

**Fig. 3:**
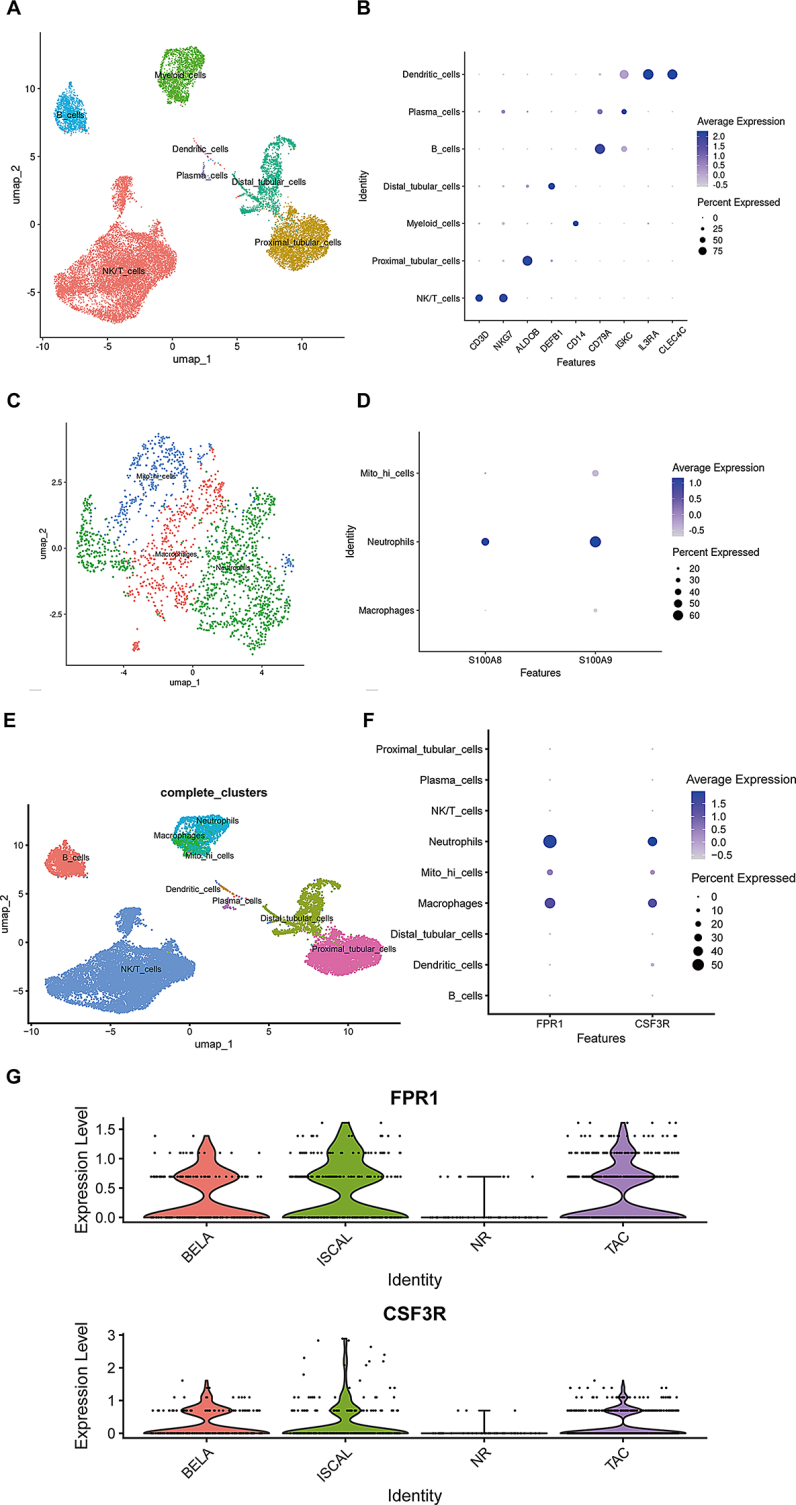
Expression levels of hub genes in the single-cell kidney transplantation dataset. (**A**) Cell subsets after dimensionality reduction clustering by UMAP. (**B**) Markers of various cell subpopulations. (**C**) Reclustering of myeloid cell populations. (**D**) Annotation of myeloid cell populations. (**E**) UMAP clustering diagram containing myeloid cell subpopulations. (**F**) The expression levels of FPR1 and CSF3R in different cell types. (**G**) The expression levels of FPR1 and CSF3R in non-rejecting and rejecting patients

We further inferred the relationship between hub genes and neutrophils by analyzing the single-cell dataset of acute rejection of kidney transplantation (Shi et al. 2023). According to the Seurat standard process (Supplementary Figs. 3 and 4), we performed UMAP clustering on the data and annotated it based on markers (Fig. 3A–B). The data includes B cells subsets, NK/T cell subsets, myeloid cell subsets, dendric cell subsets, plasma cell subsets, distal tubular cell subsets and proximal tubular subsets. Subsequently, we performed re-clustering and annotation of the myeloid cell population, which primarily includes neutrophils, macrophages, and mito-hi cells (Fig. 3C–D). We found that FPR1 and CSF3R are highly expressed in neutrophils (Fig. 3E-F). Compared to patients who experience renal transplant rejection following medication, the expression levels of FPR1 and CSF3R are diminished in non-rejecting patients (Fig. 3G). Meanwhile, we conducted an analysis of the transcriptomic data from patients who experienced rejection following kidney transplantation and those who did not (Verma et al. 2020).

### FPR1 is identified as a key gene associated with neutrophils in kidney transplantation

**Fig. 4:**
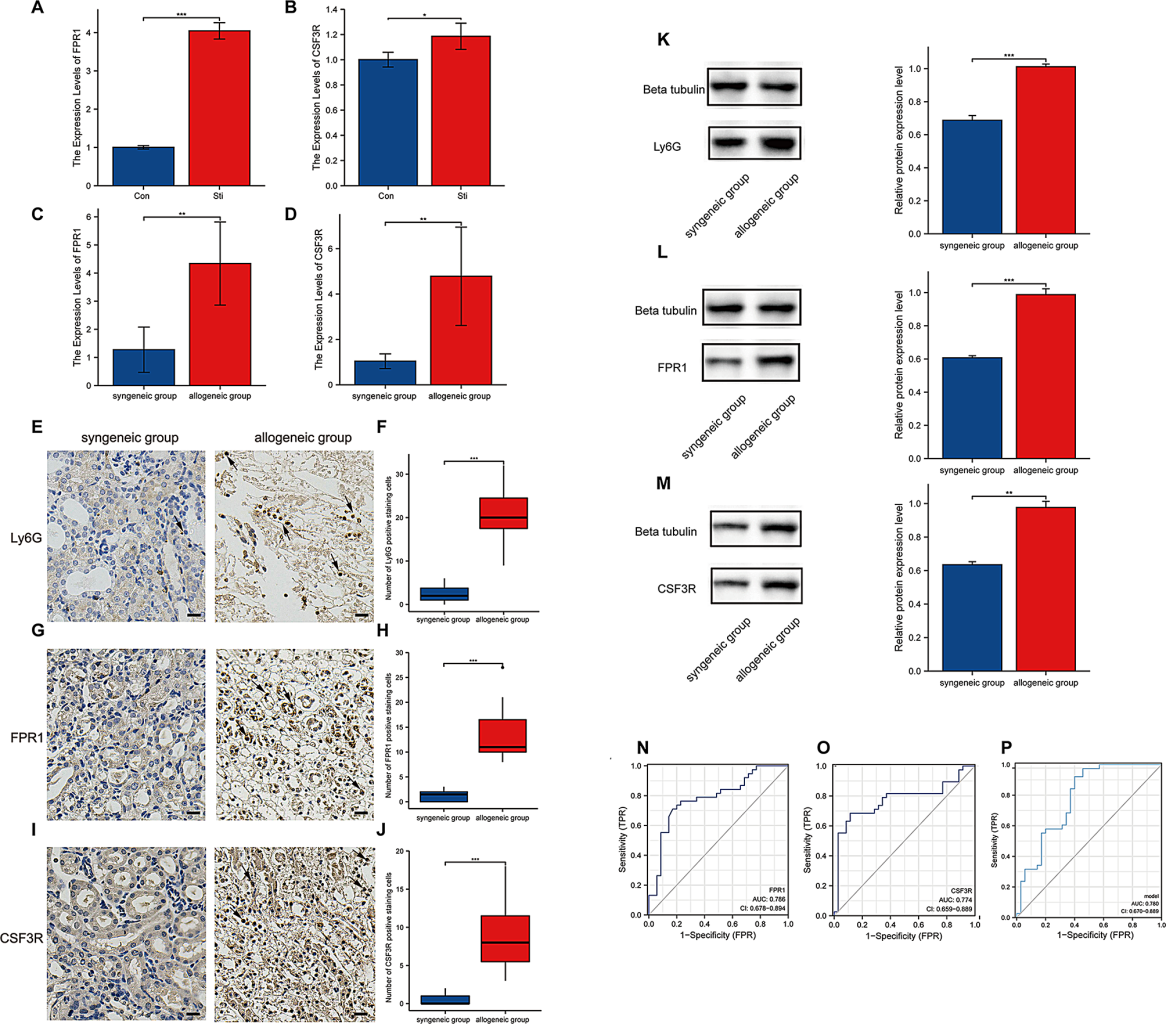
Expression levels of key genes in neutrophils and kidney grafts. (**A**) mRNA expression levels of FPR1 in neutrophils. (**B**) mRNA expression levels of CSF3R in neutrophils. (**C**) mRNA expression levels of FPR1 in kidney tissues. (**D**) mRNA expression levels of CSF3R in kidney tissues. (**E**) Detection of Ly6G expression levels in kidney tissue of the syngeneic group and allogeneic group by immunohistochemical staining. (**F**) Statistical plots of Ly6G expression levels in the syngeneic and allogeneic groups. (**G**) Detection of FPR1 expression levels in kidney tissue of the syngeneic group and allogeneic group by immunohistochemical staining. (**H**) Statistical plots of FPR1 expression levels in the syngeneic and allogeneic groups. (**I**) Detection of CSF3R expression levels in kidney tissue of the syngeneic group and allogeneic group by immunohistochemical staining. (**J**) Statistical plots of CSF3R expression levels in the syngeneic and allogeneic groups. (**K**) Protein expression levels of Ly6G in the syngeneic and allogeneic groups. (**L**) Protein expression levels of FPR1 in the syngeneic and allogeneic groups. (**M**) Protein expression levels of CSF3R in the syngeneic and allogeneic groups. (**N**) The ROC diagnostic curves of FPR1. (**O**) The ROC diagnostic curves of CSF3R. (**P**) The ROC curve of the combined diagnosis of FPR1 and CSF3R. The black arrows show the positive staining cells. Scale bar, 53 μm. *n* = 5, ns, *p* ≥ 0.05; *, *p* < 0.05; **, *p* < 0.01; ***, *p* < 0.001

In order to verify the actual expression of the above-mentioned differential genes FPR1 as well as CSF3R, neutrophils were isolated from the bone marrow of C57BL/6 mice, and the purity of the extracted neutrophils was tested by flow cytometry. The expression levels of FPR1 and CSF3R in the Sti group were statisticaly higher than those in the Con group (*p* < 0.05) (Fig. 4A-B). Then we constructed homozygous as well as heterozygous renal transplantation models in mice and obtained kidney tissues for examination. The mRNA expression levels of FPR1 as well as CSF3R were higher in the heterozygous group compared to the homozygous group (Fig. 4C-D). Immunohistochemical results showed increased infiltration of LY6G-positive cells in the homozygous group (Fig. 4E-F) and increased infiltration of FPR1- and CSF3R-positive cells in the heterozygous group (Fig. 4G-J). Western blotting results showed that the protein expression levels of Ly6G, FPR1, and CSF3R were increased in allogeneic group compared to syngeneic group (Fig. 4K-M).

Then we tested the diagnostic efficacy of FPR1 and CSF3R (Fig. 4N-P). We performed ROC analysis on FPR1 and CSF3R to verify whether they were effective markers for judging rejection after renal transplantation. The AUC value of FPR1 was 0.786 (95% CI 0.678–0.894). The AUC value of CSF3R was 0.774 (95% CI 0.659–0.889). Moreover, the AUC value for combined FPR1 and CSF3R reached 0.780 (95% CI 0.670–0.889). Therefore, compared to several other ROC analysis methods, the ROC curve of FPR1 had the capability to diagnose rejection with better specificity and sensitivity.

### Blocking FPR1 affects iron levels and MAPK pathways in neutrophils

NETs are critical for neutrophil function (Hilscher and Shah 2020; Hakkim et al. 2011), and NETs are involved in the pathology of many organ transplants, including kidney transplantation (Torres-Ruiz et al. 2020; Liu et al. 2022; Bonneau et al. 2022), whereas both FPR1 and iron metabolism can affect neutrophil function and the formation of neutrophil extracellular traps (Wu et al. 2021; Kuley et al. 2021; Kuźmicka et al. 2022; Li et al. 2022a, b; Chilingaryan et al. 2022), and changes in iron levels in many diseases affect ERK1/2 pathway activation levels (Tangudu et al. 2019; Tan et al. 2022; Bautista et al. 2016). Therefore, based on the potential intrinsic link between iron and FPR1 in neutrophils and extra-neutrophilic traps, we focused on the effect of FPR1 on iron metabolism in neutrophils.

**Fig. 5:**
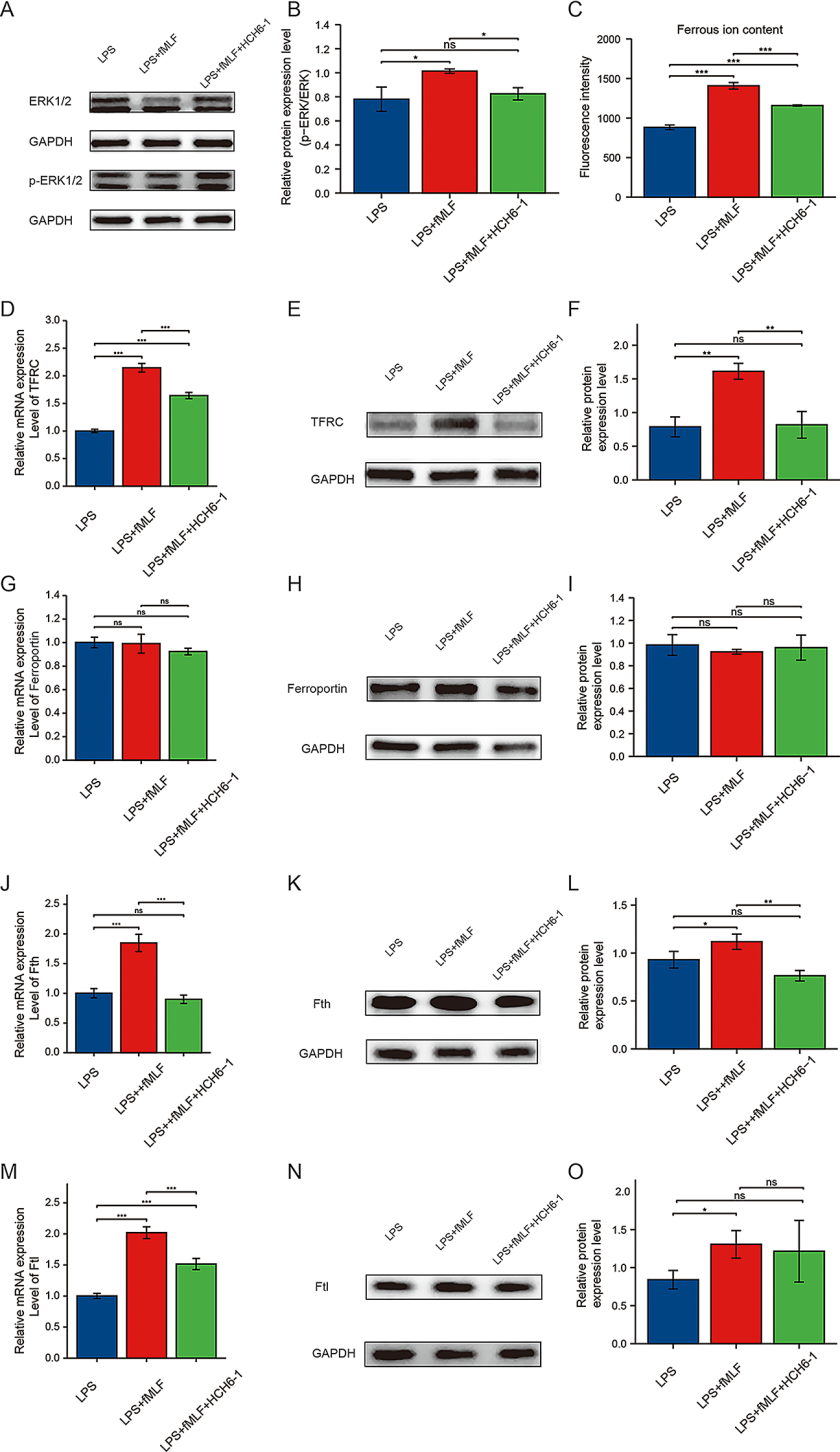
Changes in ERK1/2 pathway and iron level in neutrophils after blocking FPR1. (**A**–**B**) Detection of the protein expression level of ERK1/2 pathway. (**C**) Expression levels of ferrous ions in neutrophils in each group. (**D**) The mRNA expression level of TFRC in neutrophils of each group. (**E**–**F**) The protein expression level of TFRC in neutrophils of each group. (**G**) The mRNA expression level of ferroportin in neutrophils of each group. (**H**–**I**) The protein expression level of ferroportin in neutrophils of each group. (**J**) The mRNA expression level of Fth in neutrophils of each group. (**K**–**L**) The protein expression level of Fth in neutrophils of each group. (**M**) The mRNA expression level of Ftl in neutrophils of each group. (**N**–**O**) The protein expression level of Ftl in neutrophils of each group. The data were analyzed by mean ± SEM. *n* = 3, *, *p* < 0.05; **, *p* < 0.01; ***, *p* < 0.001

Analysis showed that the activation of ERK1/2 pathway increased in LPS-stimulated neutrophils in the presence of fMLF. In this state, the addition of HCH6-1 to block FPR1 could reduce the activation of ERK1/2 pathway of neutrophils (Fig. 5A and B). The number of ferrous ions in the neutrophils was then measured. It was found that the content of ferrous ions increased in LPS + fMLF group. After FPR1 blocking, the content of ferrous ions decreased (Fig. 5C). Iron metabolism-related indexes in neutrophils were assessed by PCR and western blotting (WB). Compared to LPS group, LPS + fMLF group had significantly higher mRNA expression levels of Transferrin receptor protein 1 (TFRC), and this trend could be inhibited by HCH6-1 (Fig. 5D). The results of WB verification were consistent with those of qPCR (Fig. 5E–F). With regard to Ferroportin 1 (Ferroportin) expressions, there is no significant difference among the three groups (Fig. 5G–I). The expression levels of mRNA and protein of Ferritin Heavy Chain (Fth) in LPS + fMLF group was greater than that in LPS group. The expression levels of Fth in LPS + fMLF + HCH6-1 group decreased after treatment with HCH6-1 (Fig. 5J–L). The mRNA expression levels of Ferritin Light Chain (Ftl) was significantly increased in LPS + fMLF group compared to LPS group, and this trend could be inhibited by HCH6-1 (Fig. 5M). The protein expression level of Ftl in LPS + fMLF + HCH6-1 group was lower than that in LPS + fMLF group, but the difference was not statistically significant (Fig. 5N–O). The expression levels of TFRC and Tfh proteins exhibited significant differences across the groups. Furthermore, we investigated the impact of LPS on the protein expression levels of TFRC and Tfh. Our findings revealed that, compared to unstimulated neutrophils, the protein expression levels of TFRC and Tfh in neutrophils stimulated with LPS were elevated (Supplementary Fig. 5).

### Effect of blocking FPR1 on neutrophil function

**Fig. 6:**
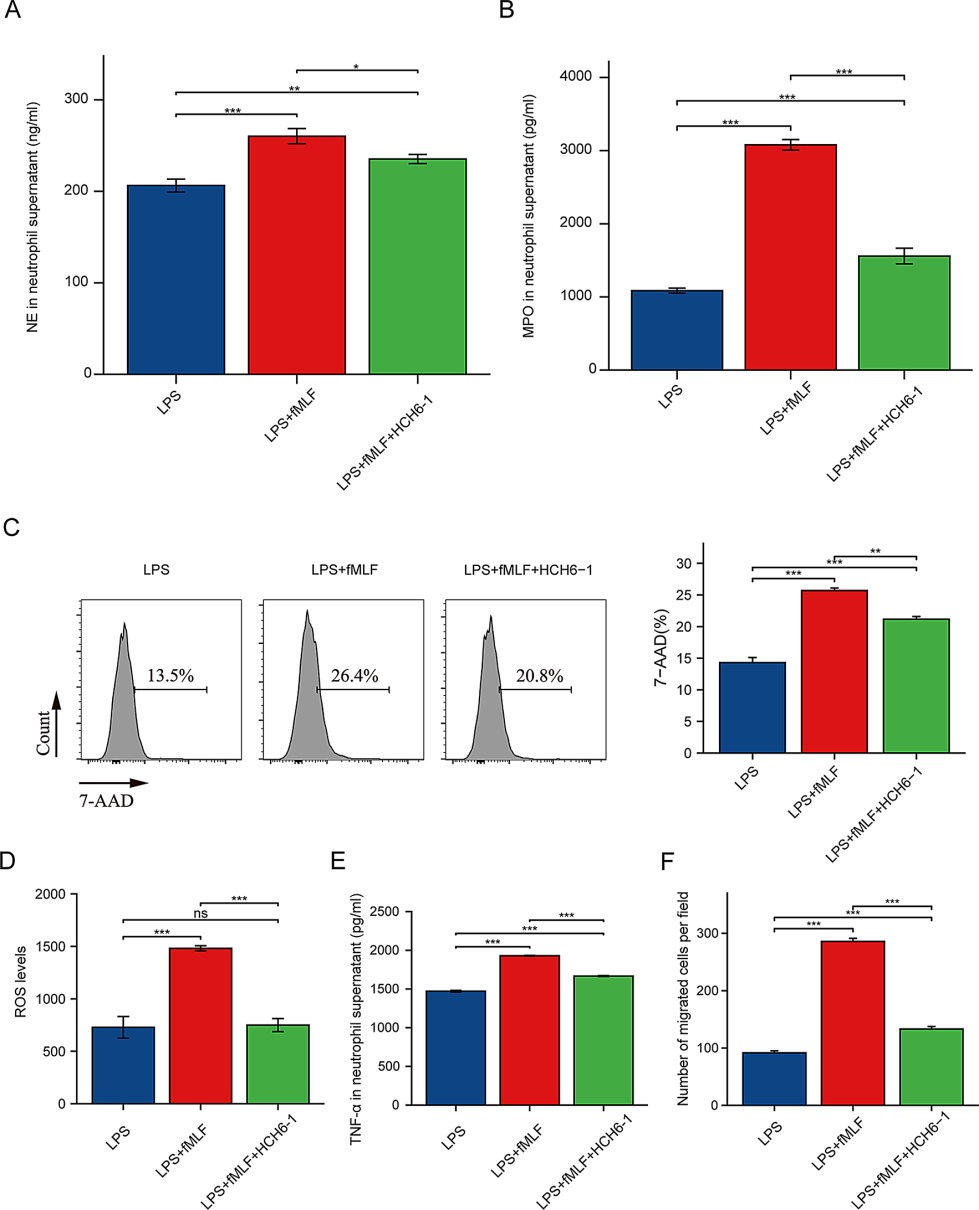
Changes in neutrophil function after blocking FPR1. (**A**) The changes in NE content in the supernatant of neutrophils in each group. (**B**) MPO content changes in the supernatant of neutrophils in each group. (**C**) The change of 7-AAD index in each group. (**D**) The changes in ROS content in neutrophils in each group. (**E**) The changes of TNF-α content in the supernatant of neutrophils in each group. (**F**) Chemotaxis of neutrophils in each group. The data were analyzed by mean ± SEM. *n* = 3, *, *p* < 0.05; **, *p* < 0.01; ***, *p* < 0.001

NETs are web-like filamentous extracellular structures of DNA, histones, and cytotoxic granule-derived proteins (Brinkmann et al. 2004; Branzk et al. 2014). Detection of MPO, NE, and extracellular DNA is an important way to assess NETs formation (Sprenkeler et al. 2022; Masuda et al. 2016). We examined NE and MPO in neutrophil supernatant. Compared with LPS group, the contents of NE and MPO in the supernatant of neutrophils in LPS + fMLF group increased, which may indicate the increased formation of NETs. After treatment with HCH6-1, the expression levels of NE and MPO in the supernatant of LPS + fMLF + HCH6-1 group decreased, but it was still higher than that of LPS group (Fig. 6A–B). And flow cytometry results showed an increase in extracellular DNA in the LPS + fMLF group, which likewise decreased after HCH6-1 treatment (Fig. 6C, Mean Fluorescence Intensity of 7-AAD in neutrophils shown in Supplementary Fig. 6). Compared to LPS group, LPS + fMLF group had significantly higher levels of intracellular ROS. After HCH6-1 treatment, the intracellular ROS level of LPS + fMLF + HCH6-1 group was significantly decreased (Fig. 6D). Consistent with the trend of ROS levels, HCH6-1 also inhibited the secretion of TNF-α by neutrophils (Fig. 6E). After LPS stimulation, neutrophils treated with fMLf could enhance the migration ability of neutrophils, and HCH6-1 could inhibit this effect (Fig. 6F).

### Blocking FPR1 alleviates acute rejection after renal transplantation in mice

**Fig. 7:**
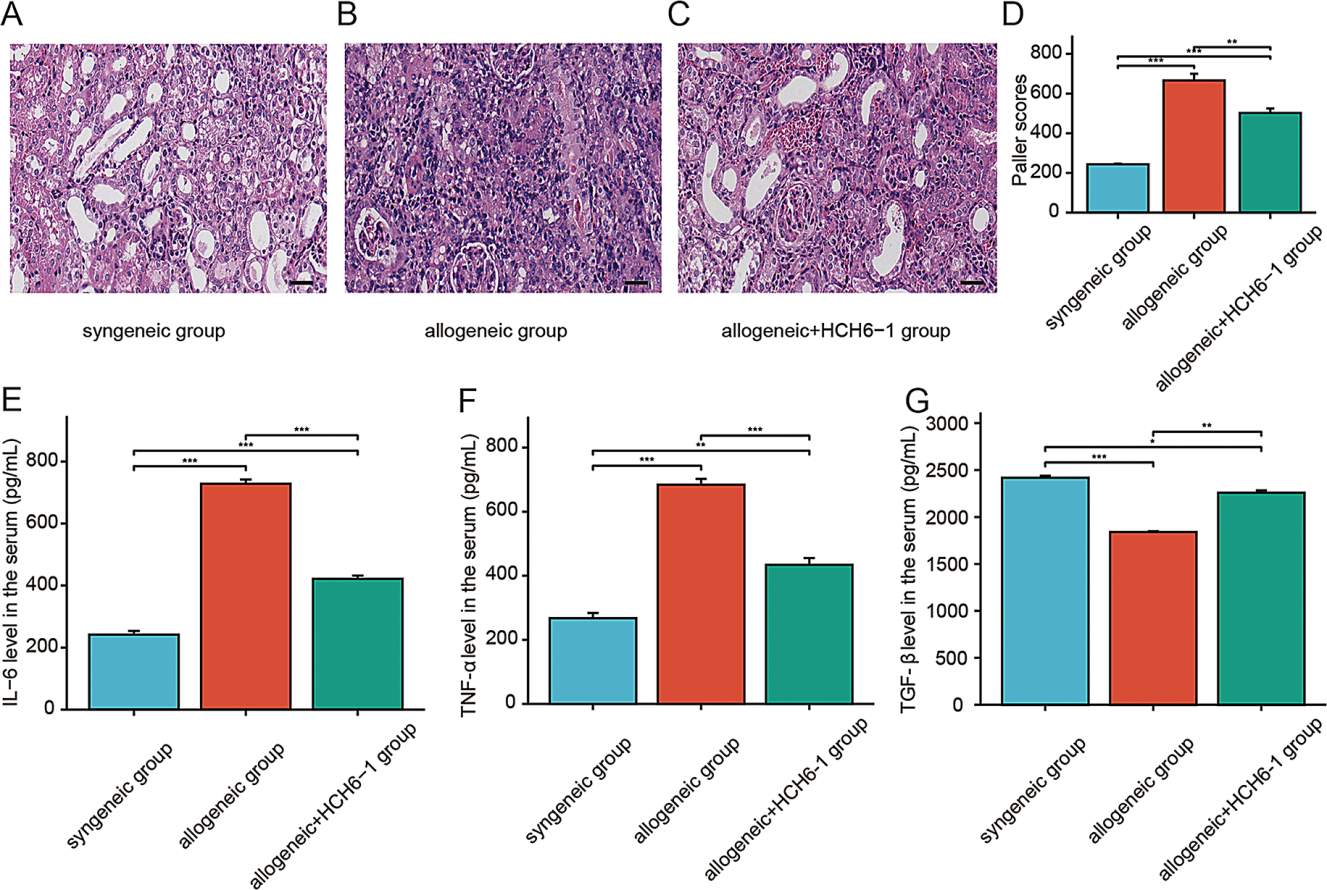
Effect of blocking FPR1 on acute rejection after renal transplantation in mice. (**A**–**C**) Injury of kidney grafts in each group of mice. (**D**) Injury scores of renal grafts in each group. (**E**) The expression level of IL-6 in the serum of mice in each group. (**F**) The expression level of TNF-α in the serum of mice in each group. (**G**) The expression level of TGF-β in the serum of mice in each group. The data were analyzed by mean ± SEM. Scale bar, 53 μm. *n* = 4, *, *p* < 0.05; **, *p* < 0.01; ***, *p* < 0.001

In the syngeneic group, the renal tubular epithelial cells slightly degenerated. Mild connective tissue hyperplasia can be seen in the local stroma. There are a small amount of dilated renal tubules in the tissue (Fig. 7A). In the allogeneic group, the tissue damage was aggravated. There are a large number of renal tubule atrophy in the kidney tissue. A large number of connective tissue hyperplasia with moderate lymphocyte infiltration can be seen in the stroma (Fig. 7B). In the allogeneic + HCH6-1 group, the renal tissue injury was alleviated after HCH6-1 treatment. The number of atrophied renal tubules decreased. The infiltration of lymphocytes in the tissue was reduced (Fig. 7C). According to the kidney injury score, after HCH6-1 treatment, kidney injury was reduced in the allogeneic group (Fig. 7D).

After that, we measured the serum cytokine levels in all groups. IL-6 and TNF-α in allogeneic group were higher than those in syngeneic group. After HCH6-1 treatment, the content of pro-inflammatory factor (IL-6, TNF-α) in the serum of allogeneic group was decreased (Fig. 7E–F). TGF-β levels were reduced in group B compared to group A. In group B, TGF-β content in serum increased after being treated with HCH6-1 (Fig. 7G).

### Alteration of neutrophil function in mouse kidney transplantation model

**Fig. 8:**
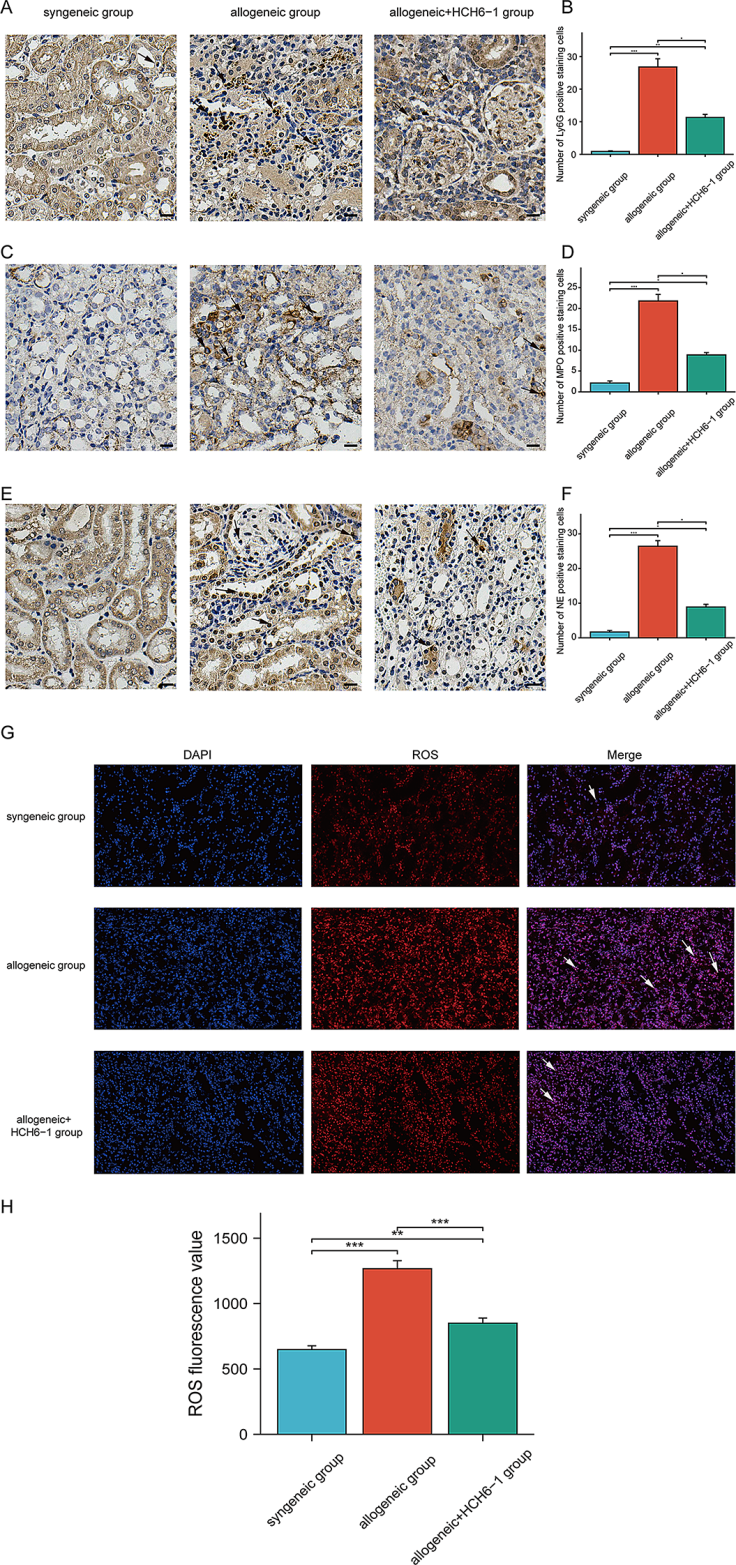
Functional expression of neutrophils in kidney transplantation. (**A**–**B**) Neutrophils in infiltration kidneys of each group. (**C**–**D**) The expression level of MPO in the kidneys of each group. (**E**–**F**) The expression level of NE in kidneys of each group. (**G**) ROS levels in the kidneys of each group. (**H**) Statistical plots of ROS levels in the kidneys of each group. The data were analyzed by mean ± SEM, *n* = 4. The arrows show the positive staining cells. Scale bar, 53 μm. *, *p* < 0.05; **, *p* < 0.01; ***, *p* < 0.001

Compared with the syngeneic group, neutrophil infiltration was increased in the allogeneic group. In the allogeneic group treated with HCH6-1, neutrophil infiltration was reduced (Fig. 8A–B). The expression levels of NE and MPO was consistent with neutrophil infiltration (Fig. 8C–F). ROS level was shown to be higher in the allogeneic group than in the syngeneic group, and ROS level decreased after HCH6-1 treatment (Fig. 8G-H).

### Proportions of T cells and iron content of neutrophils in the spleen of mouse kidney transplantation model

**Fig. 9:**
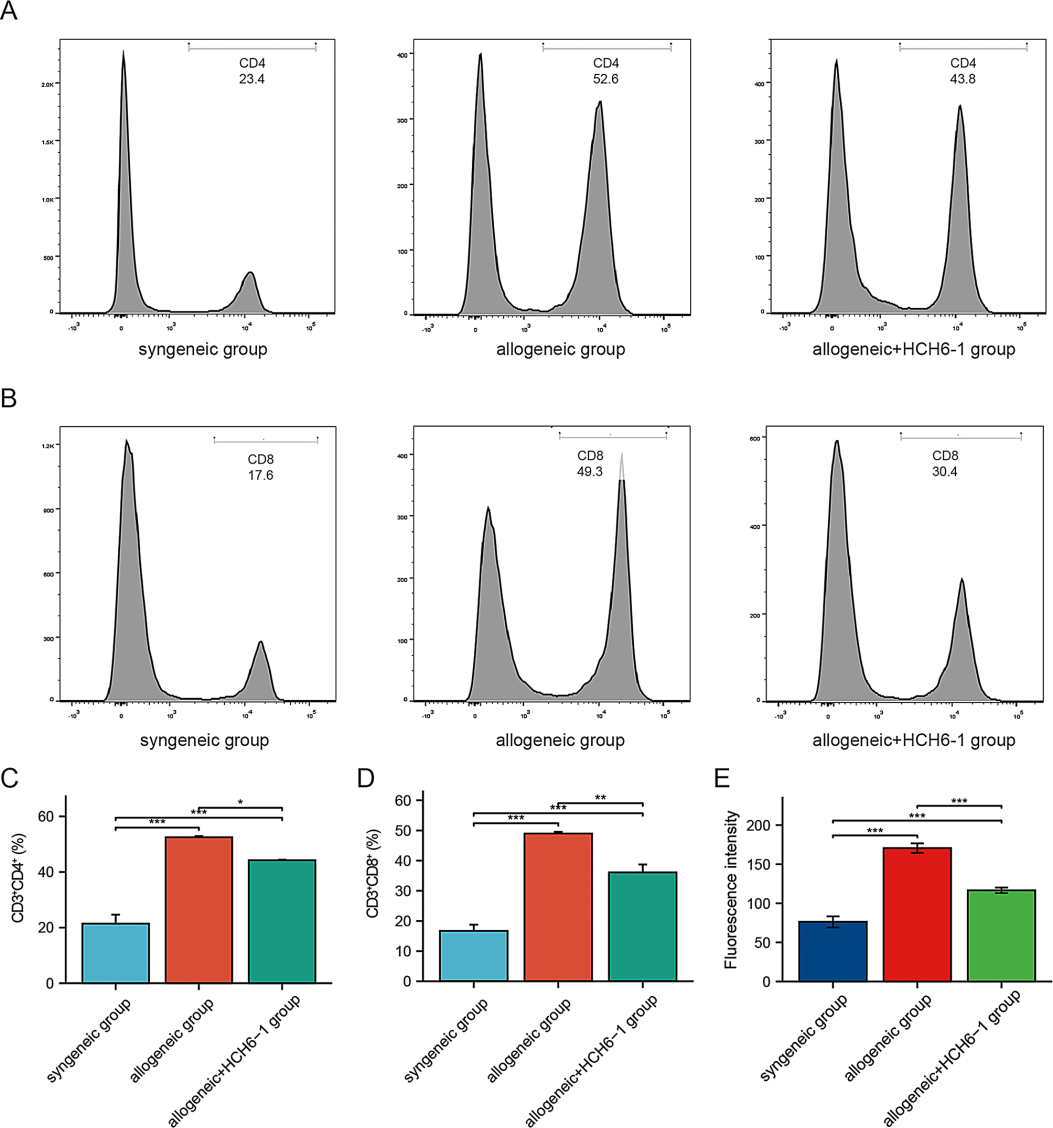
T cell proportions and iron content in neutrophils in the spleen of mice in each group. (**A**) Proportions of CD4^+^ T cells in spleens of each group. (**B**) Proportions of CD8^+^ T cells in spleens of each group. (**C**) Statistical analysis of the proportion of CD4^+^ T cells in the spleen of each group. (**D**) Statistical analysis of the proportion of CD8^+^ HCHT cells in the spleen of each group. (**E**) Ferrous ion content in neutrophils in mice of each group. The data was analyzed by mean ± SEM, *n* = 4. *, *p* < 0.05; **, *p* < 0.01; ***, *p* < 0.001

Mouse spleen T cell content was analyzed by flow cytometry, and it was revealed that the allogeneic group had a greater CD4^+^ T cell content than the syngeneic group. CD4^+^T cell content in allogeneic + HCH6-1 group was lower than that of the allogeneic group (Fig. 9A-C). The proportion of CD8^+^T cells was consistent with that of CD4^+^T cells (Fig. 9B-D).

Afterward, the content of ferrous ions in neutrophils in mice was detected. The content of ferrous ions in the allogeneic group was higher than that in the syngeneic group. After the allogeneic group was treated with HCH6-1, the content of ferrous ions in neutrophils decreased (Fig. 9E).

## Discussion

Neutrophils are the main immune cells in stable blood circulation (Mestas and Hughes 2004). Meanwhile, neutrophils are a recognized marker of graft injury (Schofield et al. 2013). Neutrophils are a population of cells that have an early response to infiltrating cells and solid organ xenografts after transplantation, and neutrophils induce tissue damage in an antibody-dependent and antibody-independent manner under xenogenic conditions (al-Mohanna et al. 1996, 1997; Sachs et al. 2007; Honda et al. 2013).

Neutrophils are typically the earliest leukocytes to infiltrate transplanted organs and serve as definitive markers of transplant injury (Schofield et al. 2013). During ischemia-reperfusion and rejection processes, damage-associated molecular patterns (DAMPs) released from necrotic cells and the extracellular matrix (ECM) induce the expression of inflammatory factors by stimulating neutrophil pattern recognition receptors (PRRs). This process promotes the infiltration of neutrophils from peripheral blood into the graft (Scozzi et al. 2017) and enhances their activation (Braza et al. 2016). In this study by performing GO as well as KEGG analysis on the GSE14346 dataset, we did find that neutrophil-mediated immune processes are strongly associated with acute rejection of kidney transplantation. In the CIBERSORT analysis, we found that the proportion of neutrophils in the AR group was higher than that in the STA group. Then, we performed WGCNA on the genes of the AR group samples and found that the brown and black modules were closely associated with neutrophils, and the pivotal genes FPR1, CSF3R, and CXCR1 were extracted using PPI network analysis.

By constructing a mouse kidney transplantation model, we verified that FPR1 as well as CSF3R were indeed highly expressed during acute rejection. The ability of FPR1 to diagnose rejection with better specificity and sensitivity was suggested by ROC analysis. Eventually FPR1 was recognized as a key gene in acute rejection of renal transplantation, in fact, it has been reported in the literature that blockade of FPR1 reduces neutrophil infiltration and function (Honda et al. 2017), based on which this paper attempted to explore the possible mechanisms by which FPR1 regulates acute rejection of renal transplantation by affecting neutrophils.

NETs are network structures formed by neutrophils, consisting of extracellular DNA, granule proteins and histones (Hilscher and Shah 2020; Hakkim et al. 2011), which are essential for neutrophil function. NETs are involved in various pathological processes in organ transplantation. NETs in the serum of patients with acute rejection after liver transplantation is closely related to the expression of liver function indicators (Liu et al. 2022). Elevated NETs in the perioperative period of lung transplantation are associated with primary graft dysfunction after human lung transplantation and can be used as a marker for disease recognition (Bonneau et al. 2022). Renal transplant biopsies show increased neutrophils as well as NET (Torres-Ruiz et al. 2020).

A correlation between FPR1 and neutrophil extracellular traps has been documented. In patients with slow plus acute liver failure, the absolute count of circulating neutrophils has been found to be increased and there is an increased ability to generate neutrophil extracellular traps, while the difference in the expression levels of FPR1 is statistically significant in patients with slow plus acute liver failure versus healthy subjects (Wu et al. 2021). In studies utilizing NETs as prognostic diagnostic markers for head and neck squamous cell carcinoma, FPR1 has been identified as a gene associated with NETs (Li et al. 2022a, b). In systemic sclerosis, it has been reported that mitochondria-derived N-formylmethionine induces excessive neutrophil activation and generation of neutrophil extracellular traps through an FPR1-dependent mechanism (Kuley et al. 2021).

Iron is an essential element for cellular activity (Andreini et al. 2018), and iron homeostasis also affects the body’s immune system, including intrinsic and adaptive immunity (Nakamura et al. 2019). Iron metabolism-related proteins are also expressed in neutrophils as essential cells for innate immunity, and iron is an important element for neutrophil functioning (Cronin et al. 2019). Iron-dependent metalloprotein MPO in neutrophils has a bactericidal impact through its Fe3^+^/Fe2^+^ redox state (Arnhold et al. 2003).

It has been shown that iron excess affects neutrophil extracellular traps and the release of reactive oxygen species (Kuźmicka et al. 2022). In ischemic stroke, the peri-infarct acid-base environment, oxygen concentration and iron ions may influence the formation of neutrophil extracellular traps (Li et al. 2022a, b). In myocardial infarction, coronary thrombi contain neutrophilic extracellular traps and iron deposition is located in the region of neutrophilic extracellular traps (Chilingaryan et al. 2022).

Based on the potential intrinsic link between iron and FPR1 in neutrophils as well as in neutrophil extracellular traps, we focused on the effect of FPR1 on iron metabolism in neutrophils, which alters neutrophil function. In vitro experiments, we found increased expression levels of TFRC and Fth in neutrophils after stimulation, indicating an increased demand for iron after cell stimulation. At the same time, the content of ferrous ions in neutrophils increased. FPR1 blocking can weaken this trend. In animal models, we also found that blocking FPR1 reduced the number of ferrous ions in neutrophils in vivo.

Changes in iron levels can affect the ERK1/2 pathway, which has been studied in many diseases, such as liver disease (Tangudu et al. 2019), diabetic nephropathy (Tan et al. 2022), and neurodegeneration (Bautista et al. 2016). In our study, the ERK1/2 pathway was activated after neutrophils were stimulated, and the ERK1/2 pathway was inhibited after blocking FPR1. Neutrophils infiltrating tissue produce ROS and pro-inflammatory cytokines (Zhai et al. 2011; Nakamura et al. 2017). In this study, we found that blocking FPR1 reduced ROS and pro-inflammatory cytokines produced by neutrophils, and we also demonstrated this phenomenon in a mouse kidney transplant model.

Our study exposed that NETs were elevated in acute rejection of kidney transplantation, and the kidney injury was aggravated. After blocking FPR1, the formation of NETs was decreased, and the kidney injury was alleviated. Therefore, blocking FPR1 may be a way to alleviate acute rejection of kidney transplantation.

T cell-mediated rejection is one of the primary effector mechanisms of organ transplant rejection and represents the most common type of acute rejection response (Loupy et al. 2020). The spleen is the largest immune organ in the body, containing a substantial number of T cells and B cells, serving as the central hub for both cellular and humoral immunity (Casciani et al. 2020; Morgan et al. 2024; Jenkins et al. 2021; Bangs et al. 2022). In the context of kidney transplantation research, splenic T cells are also considered an important reference indicator for observing immune rejection responses (Zimmerer et al. 2022; Rosales et al. 2022; Zhang et al. 2021). And studies have shown that there is interaction and communication between T cells and neutrophils (Muller et al. 2009). Infiltrating neutrophils may produce cytokines that mediate T-cell migration (Kraemer et al. 2022; Borregaard et al. 2007). Meanwhile, neutrophils secrete numerous cytokines and proteases that enhance T cell proliferation and cytokine secretion, hence enhancing the adaptive immune response (Bretz et al. 1976; Tani et al. 2001). NETs are an effective means of containing and eliminating pathogens because they not only eliminate microbes but also prevent incidental tissue injury (Papayannopoulos and Zychlinsky 2009; Belorgey and Bieth 1995). Studies have shown that NETs can indirectly affect T cell function by activating dendritic cells (Garcia-Romo et al. 2011; Lande et al. 2011; Means et al. 2005; Barrat et al. 2005). Additionally, NETs can activate T cells by lowering their activation threshold, thereby enhancing the ability of adaptive immune responses (Tillack et al. 2012). These studies showed that neutrophils and NETs were closely related to T cells. In this study, blocking FPR1 reduced proportion of CD4^+^ and CD8^+^ T cells in acute rejection animal models. Its internal mechanism needs further study.

## Conclusions

This study investigated the role of neutrophils in kidney transplant rejection by analyzing a dataset of patients with and without rejection after kidney transplantation. This study found that FPR1 modulates neutrophil function and affects neutrophil iron metabolism, and the blockade of FPR1 alleviated damage to the renal graft. This study provides interesting new insights into a possible role of FPR1 in neutrophils in transplant rejection and highlights new possible targets for immunomodulatory strategies for clinical treatment.

## Electronic supplementary material

Below is the link to the electronic supplementary material.

Supplementary Material 1

Supplementary Material 2

Supplementary Material 3

Supplementary Material 4

Supplementary Material 5

## Data Availability

The datasets generated during the current study are available in the NCBI database. Relevant information has been provided in the Methods and Materials section.

## Abbreviations

AR: Acute rejection
AUC: Area under the ROC curve
DEGs: Differentially expressed genes
ELISA: Enzyme-linked immunosorbent assay
FPR1: Formyl peptide receptor 1
GEO: Gene Expression Omnibus
GO: Gene Ontology
GPC: G-protein-coupled receptor
GSEA: Gene Set Enrichment Analysis
KEGG: Kyoto Encyclopedia of Genes and Genomes
NETs: Neutrophil extracellular traps
PPI: Protein-protein interaction
RT-PCR: Real-time polymerase chain reaction
TFRC: Transferrin receptor protein
TOM: Topological overlap matrix
WGCNA: Weighted gene coexpression network analysis
ROS: Reactive oxygen species
