# Supplementary figures and images for: FPR1 affects acute rejection in kidney transplantation by regulating iron metabolism in neutrophils
Source: Mol Med. 2025 Jan 23;31:23. doi: 10.1186/s10020-025-01077-w (PMC11758745; doi:10.1186/s10020-025-01077-w)

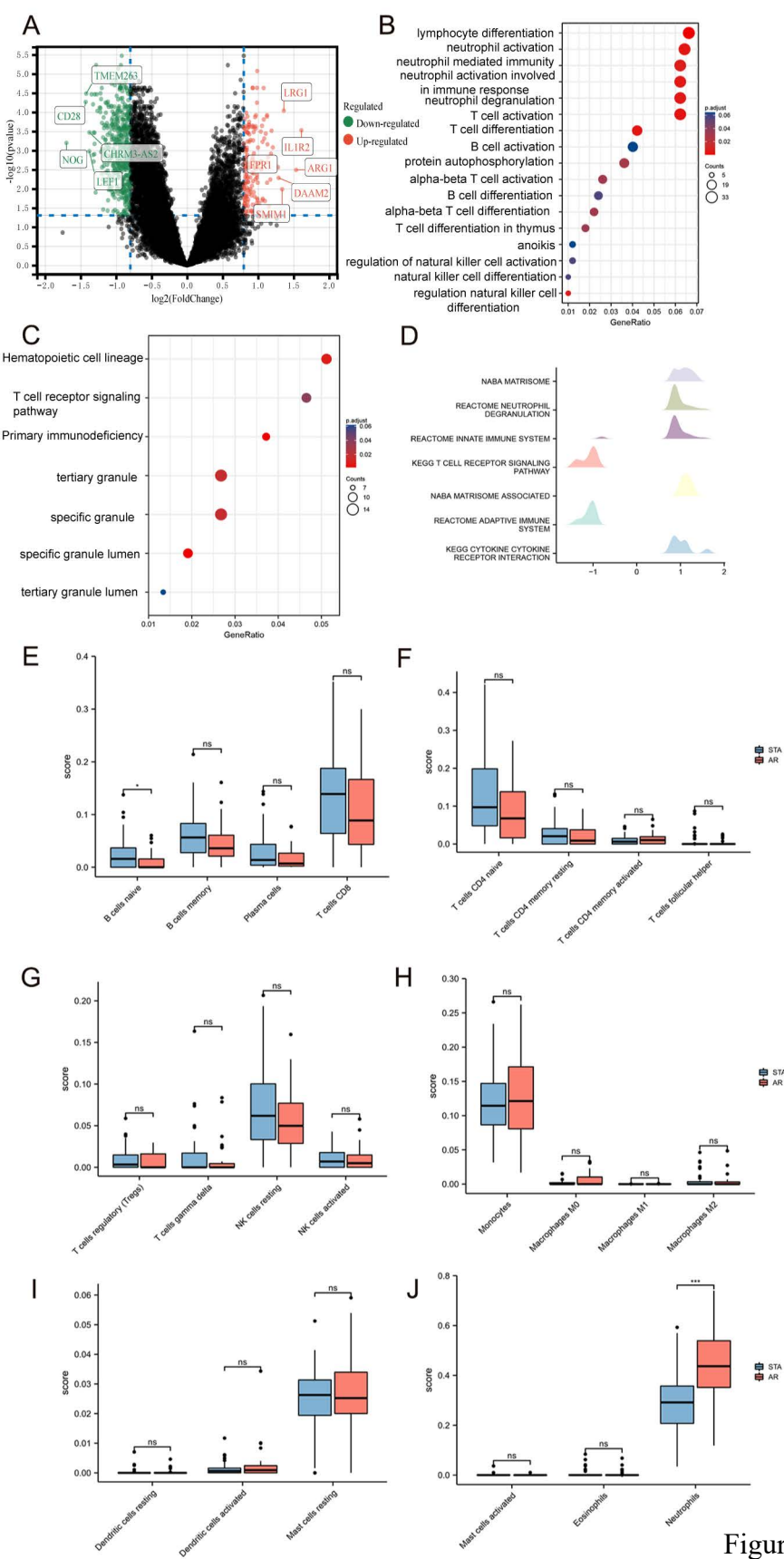

Figure 1

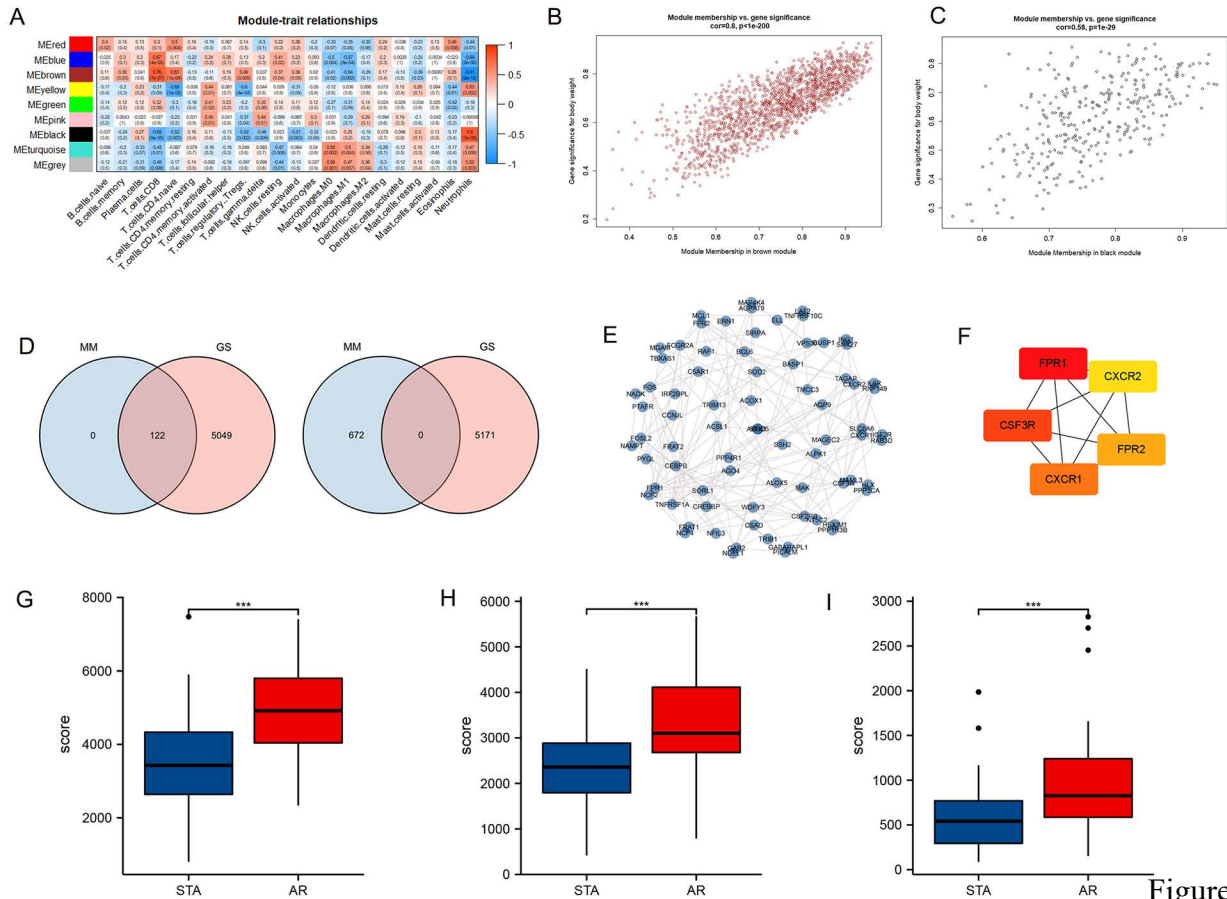

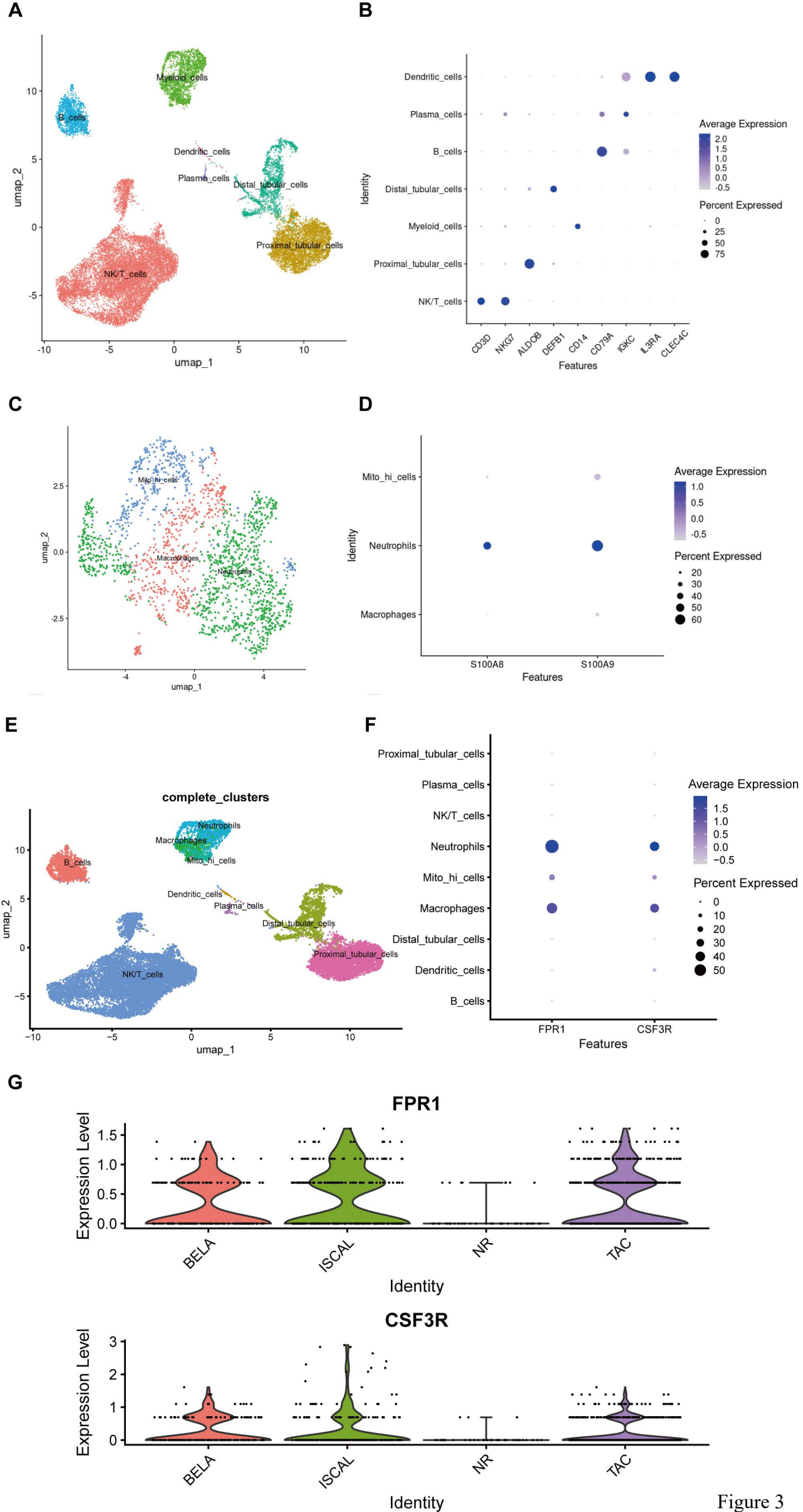

Figure 3

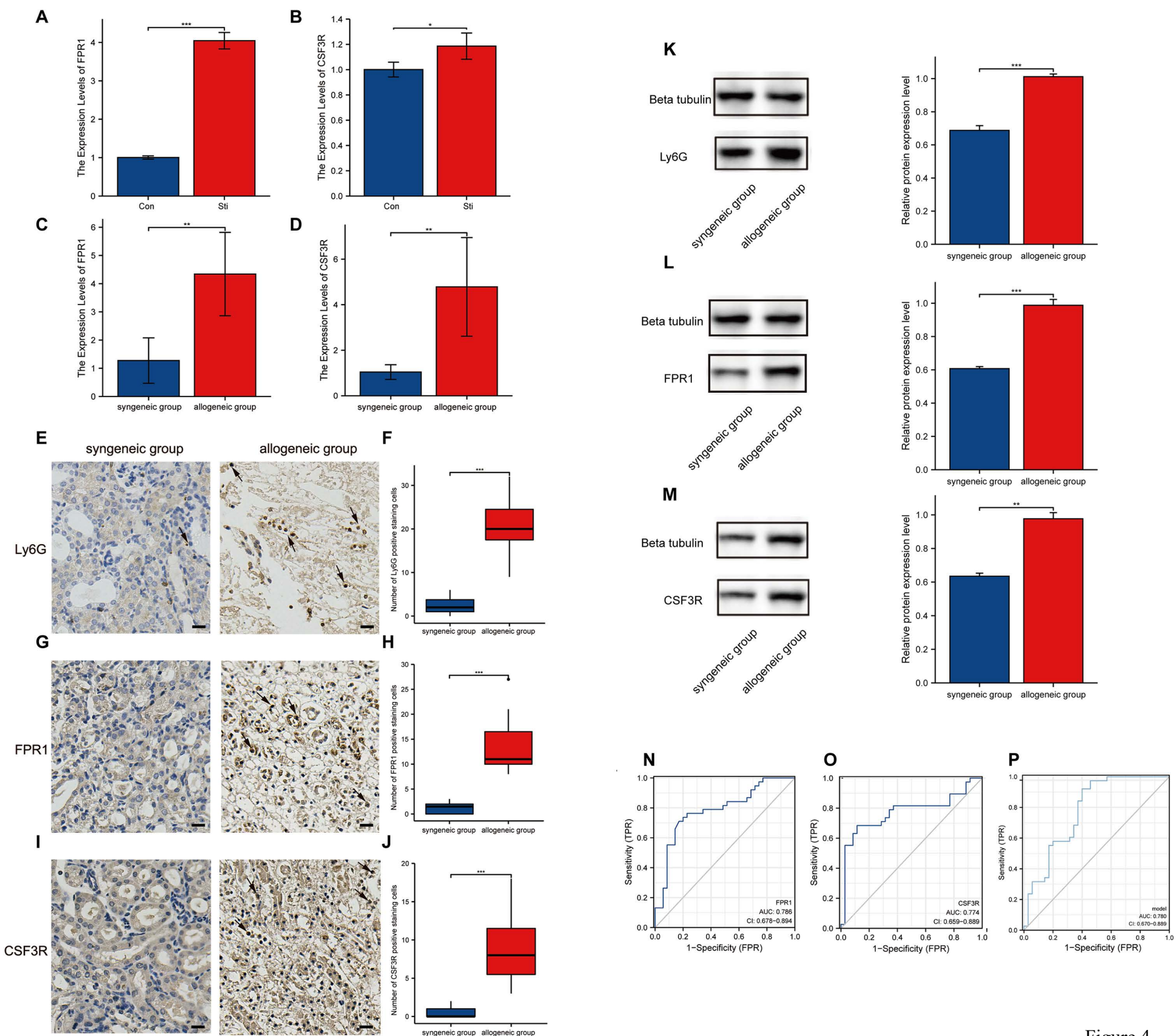

Figure 4

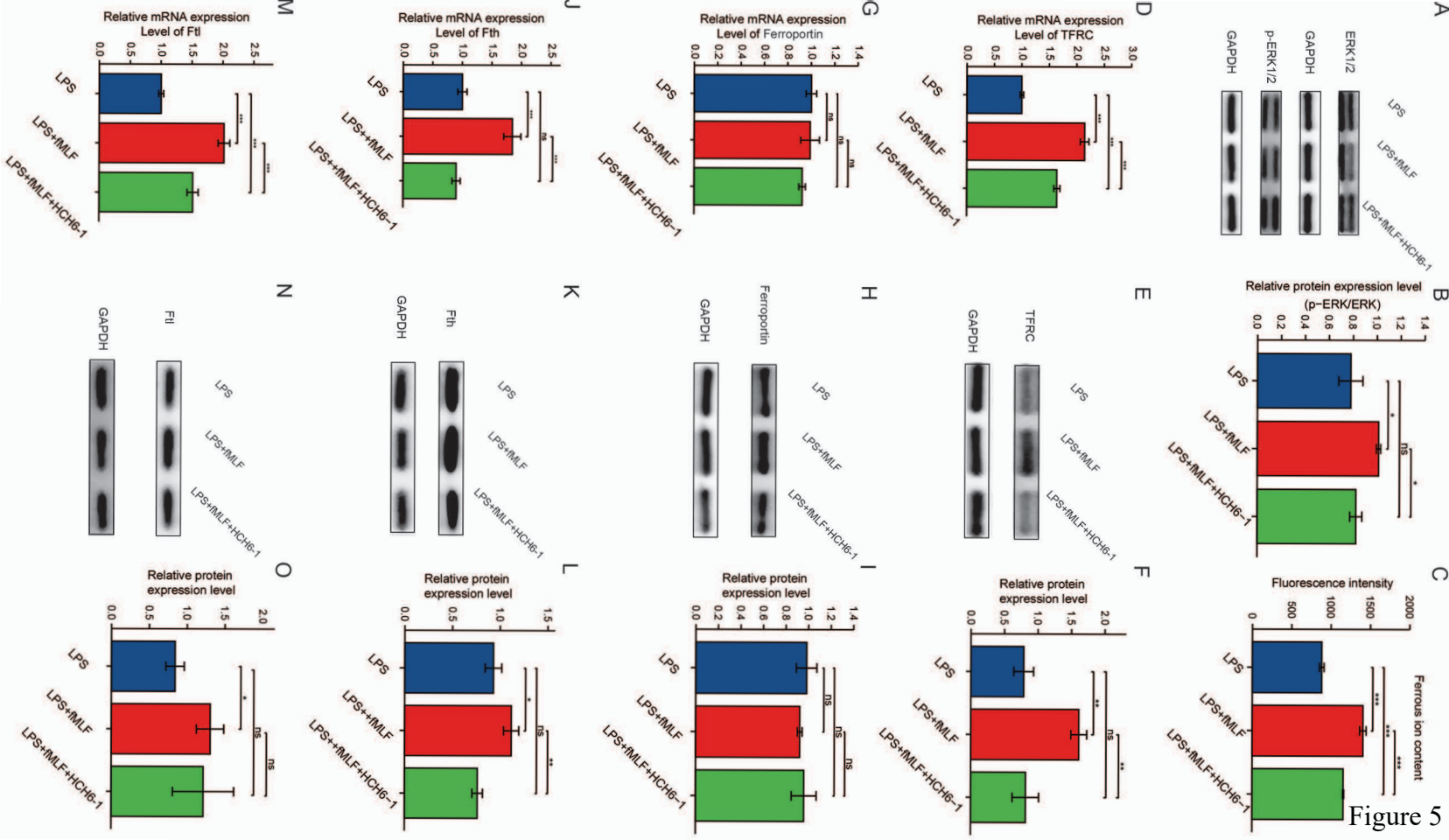

Figure 5

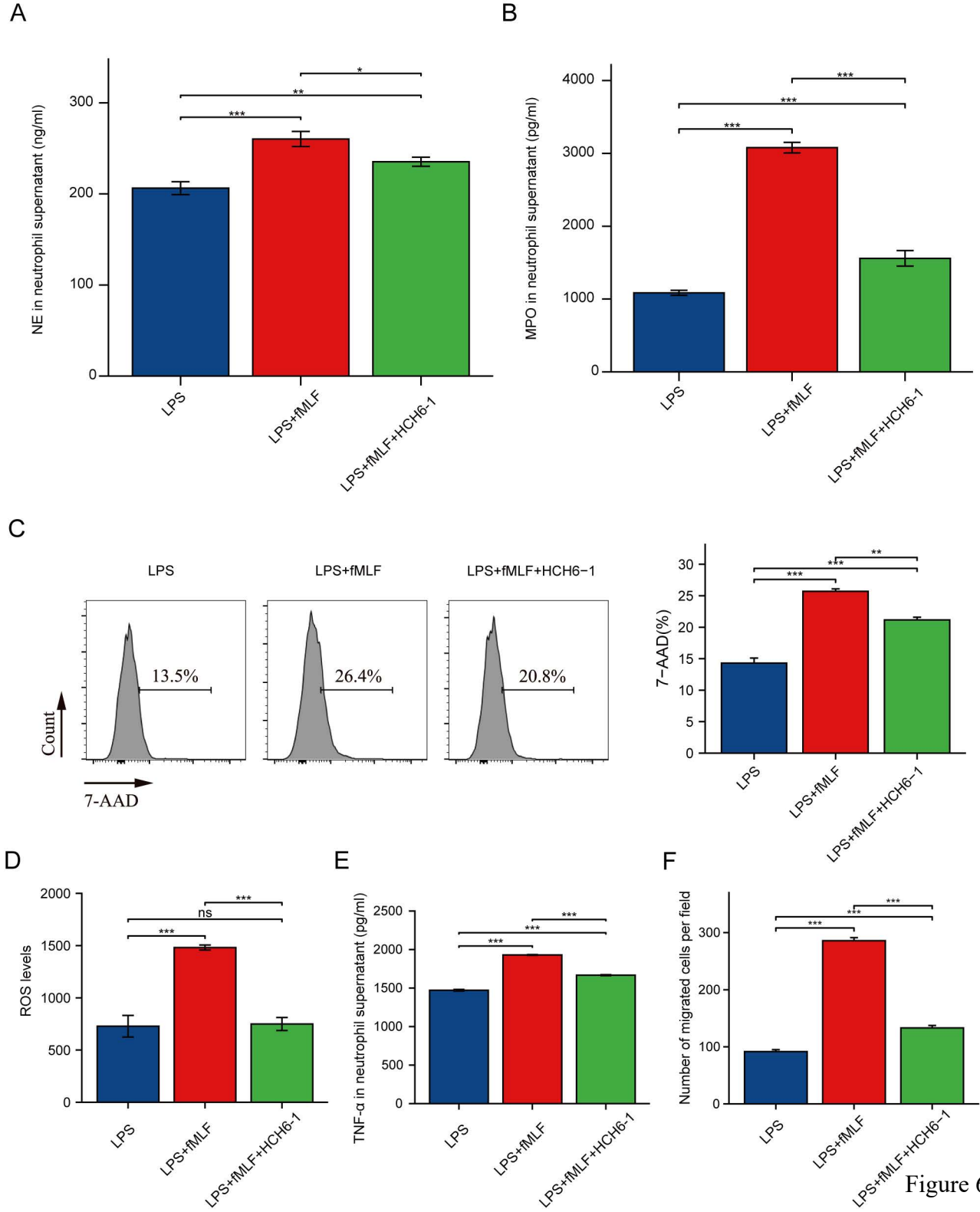

Figure 6

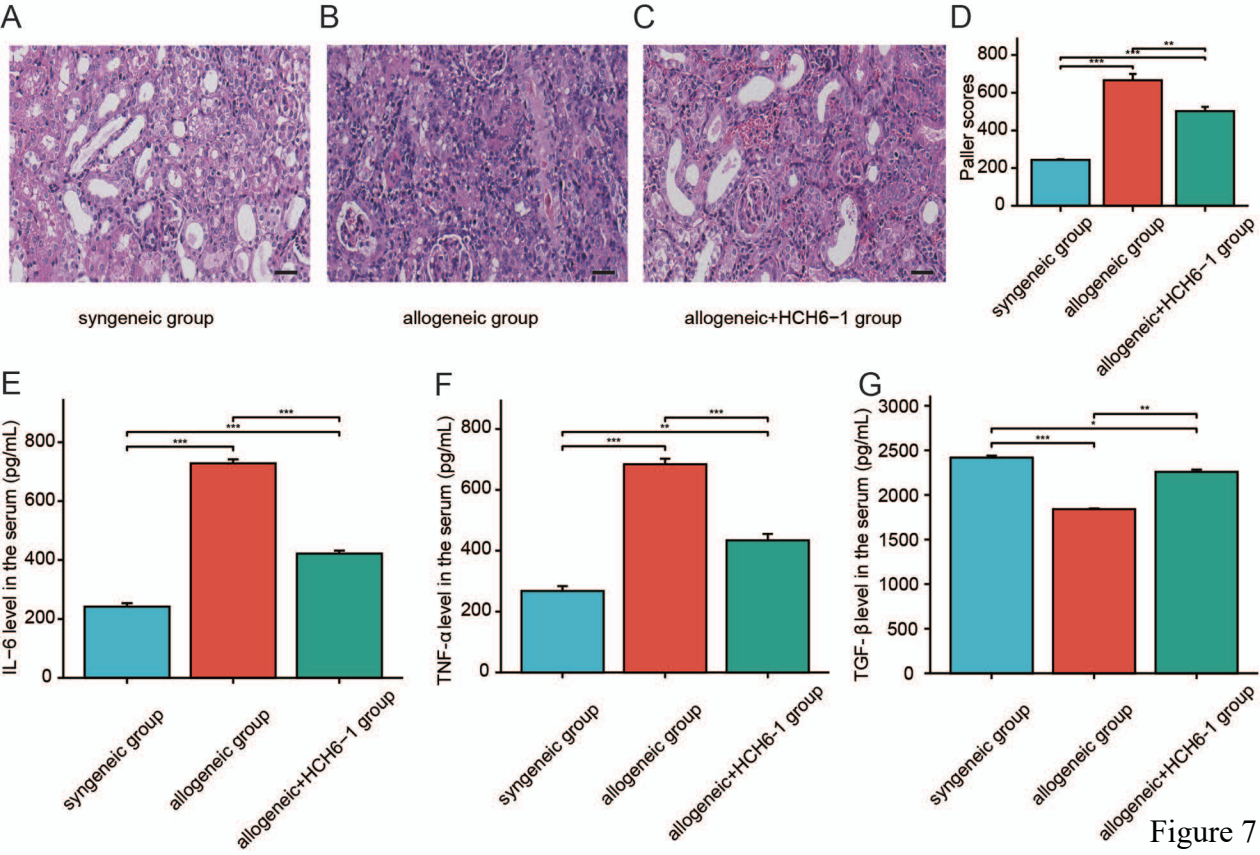

Figure 7

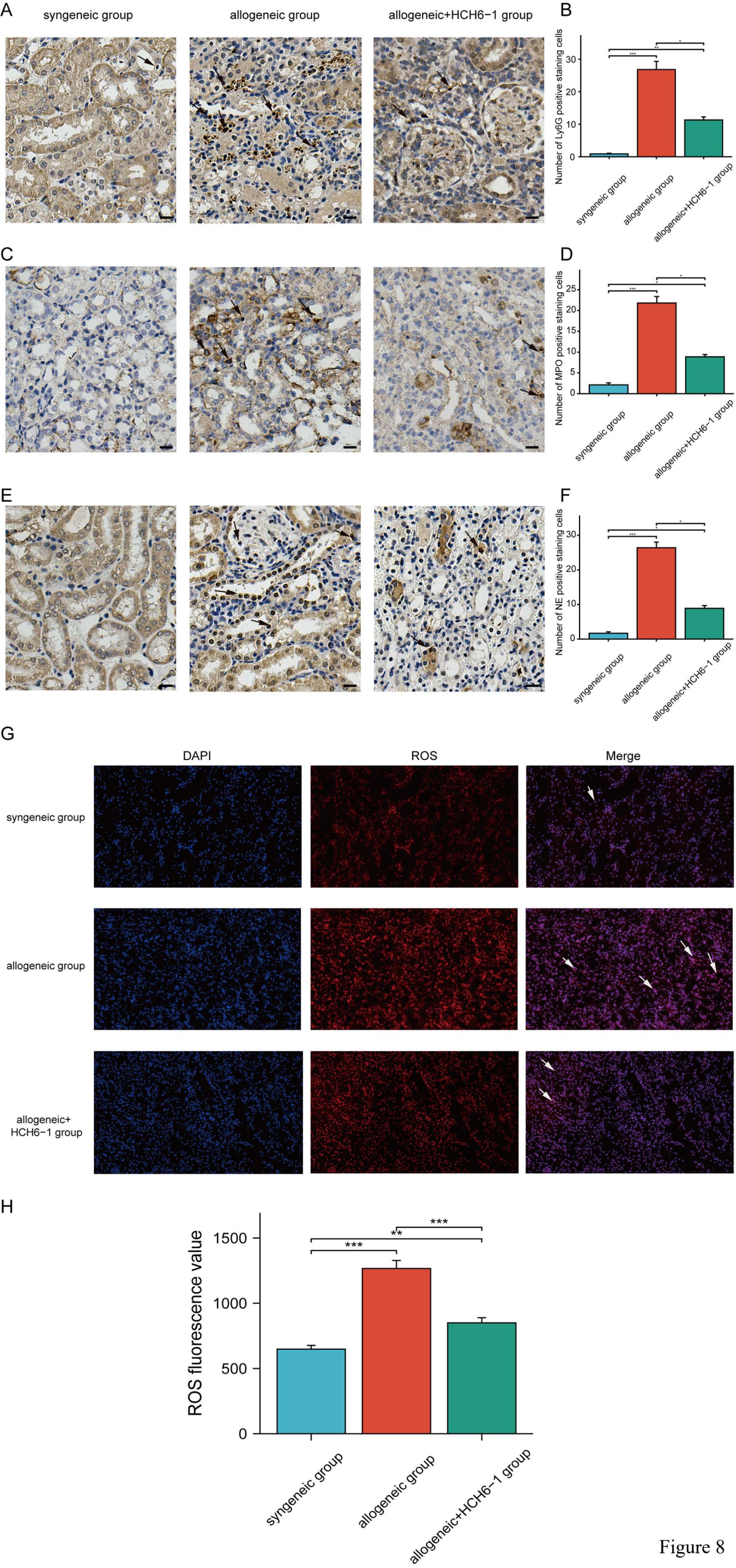

Figure 8

A

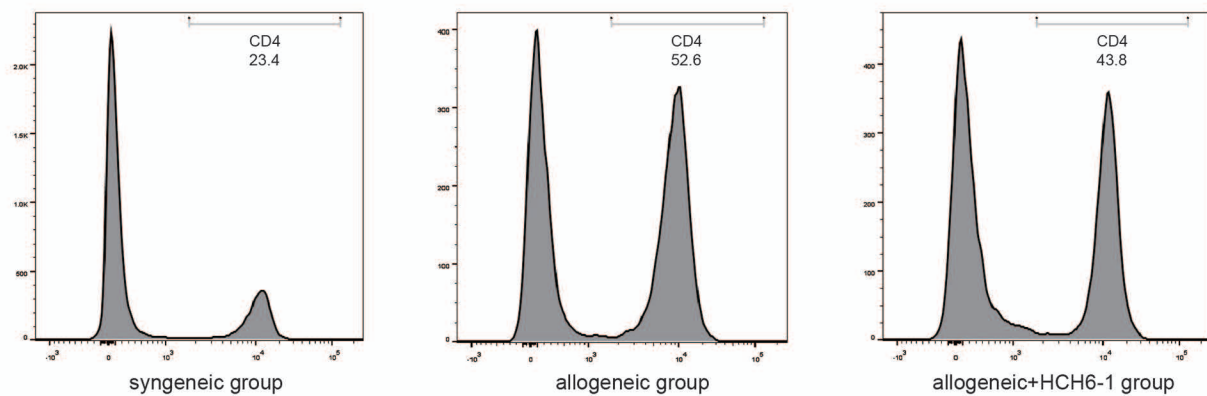

B

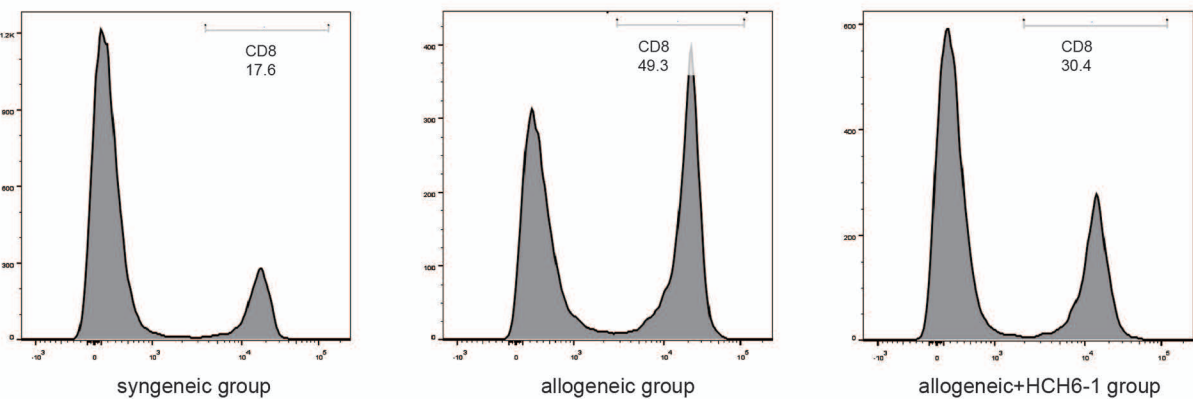

C

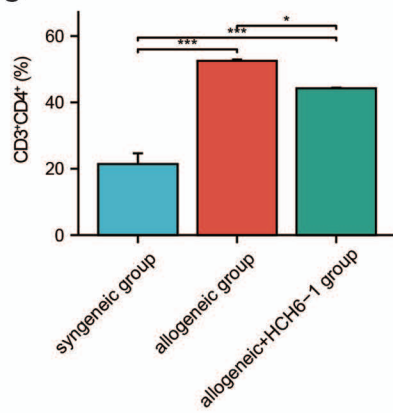

D

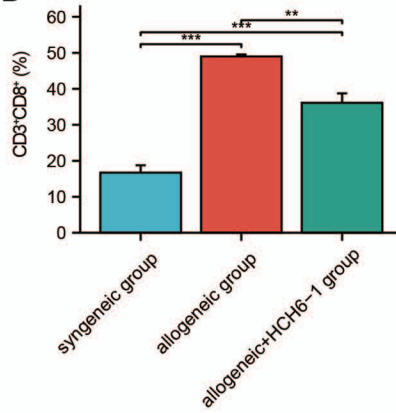

E

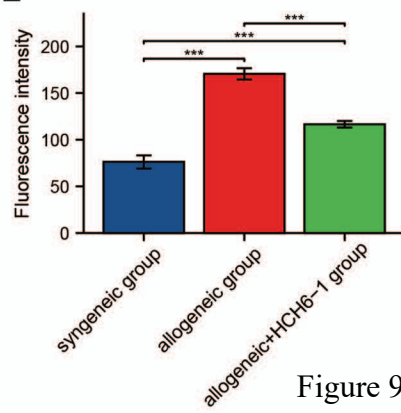

Figure 9

Supplement: Supplementary file 1 — Supplementary Material 1 [file 10020_2025_1077_MOESM1_ESM.pdf]
