# Supplementary material for: FPR1 affects acute rejection in kidney transplantation by regulating iron metabolism in neutrophils
Source: Mol Med. 2025 Jan 23;31:23. doi: 10.1186/s10020-025-01077-w (PMC11758745; doi:10.1186/s10020-025-01077-w)
Supplement: Supplementary file 3 — Supplementary Material 3 [file 10020_2025_1077_MOESM3_ESM.docx]

**
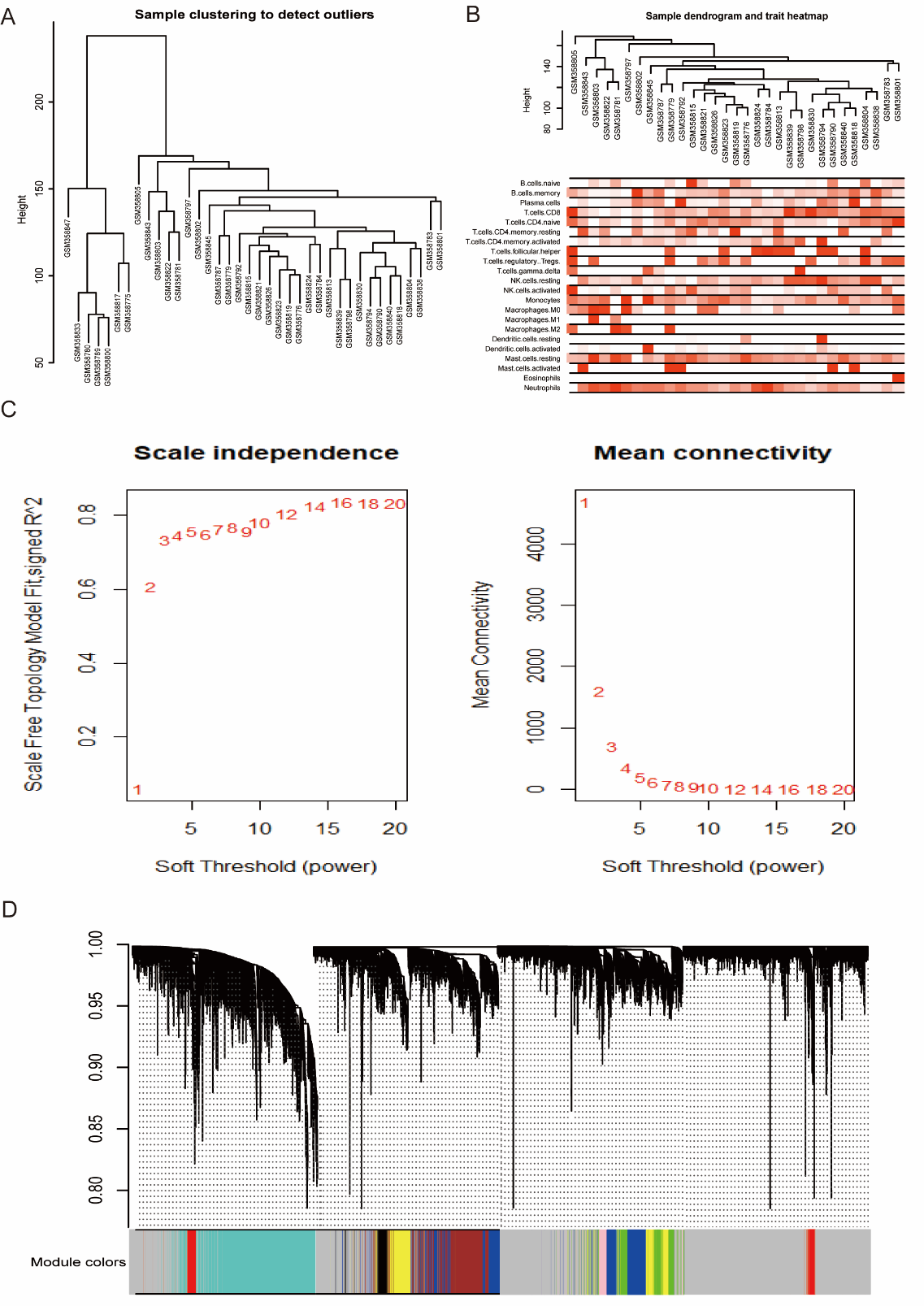
**

**Supplementary Figure 1: Results of the WGCNA process.**

(A) Clustering of dataset samples. (B) Clustering of samples and immune cell characteristics. Color intensity indicates the correlation of the immune cell to the sample, and the darker the color is, the stronger the correlation. (C) Analysis of the scale-free fit index (left) and the mean connectivity (right) for various soft-thresholding powers. (D) Dendrogram of genes clustered based on a dissimilarity measure.


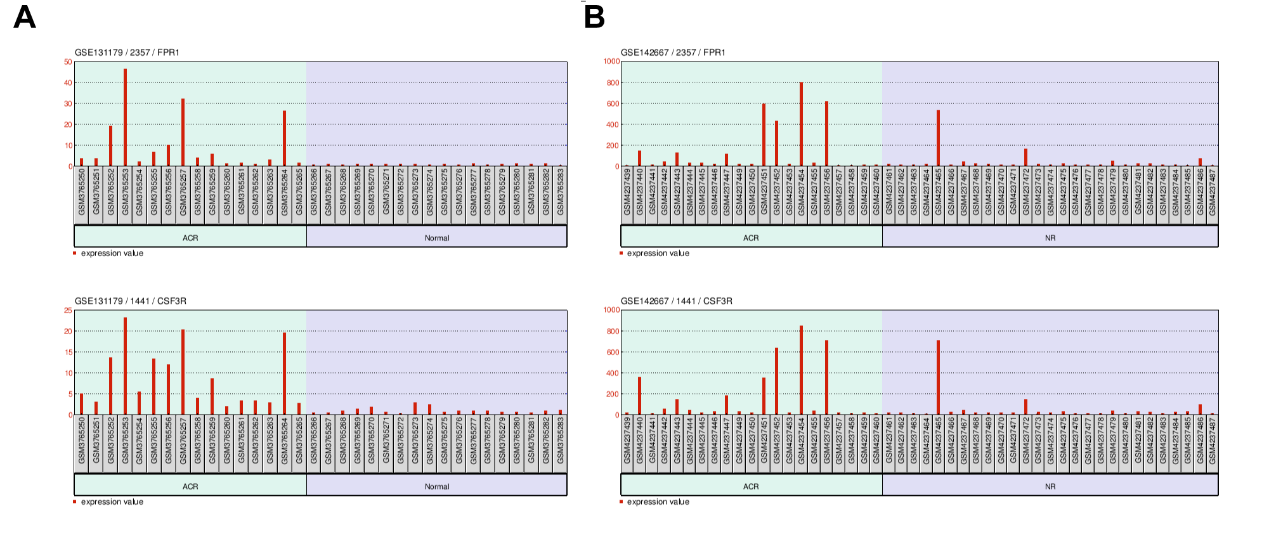


**Supplementary Figure** **2: Transcriptional sequencing of kidney-related samples from non-rejecting and rejecting patients.**

(A) Expression levels of FPR1 and CSF3R in renal tissues of non-rejected and rejected patients undergoing kidney transplantation. (B) Expression Levels of FPR1 and CSF3R in the urine of Non-Rejected and Rejected Patients following Kidney Transplantation.


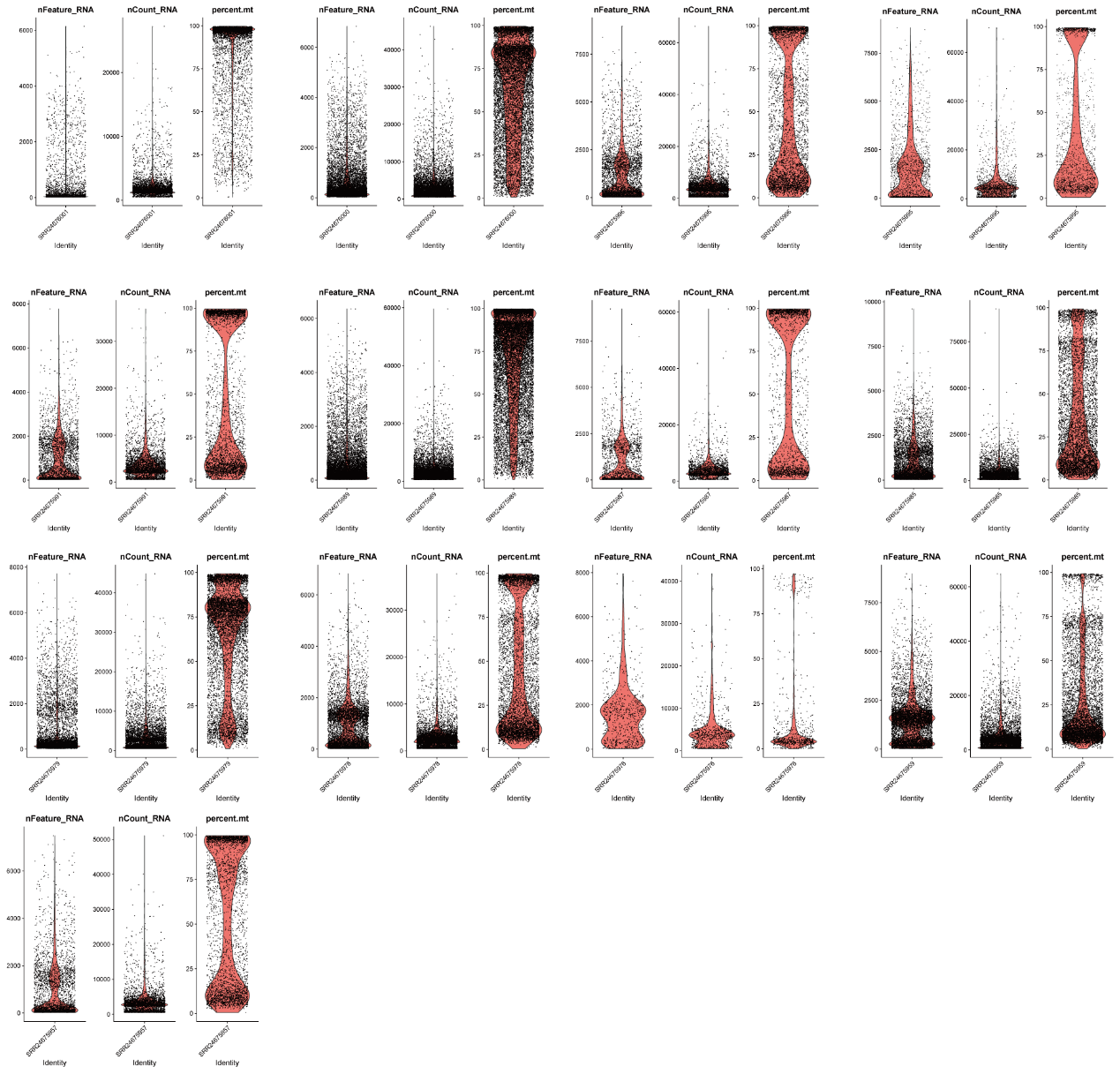


**Supplementary Figure** 3 **Quality Control of Datasets.**


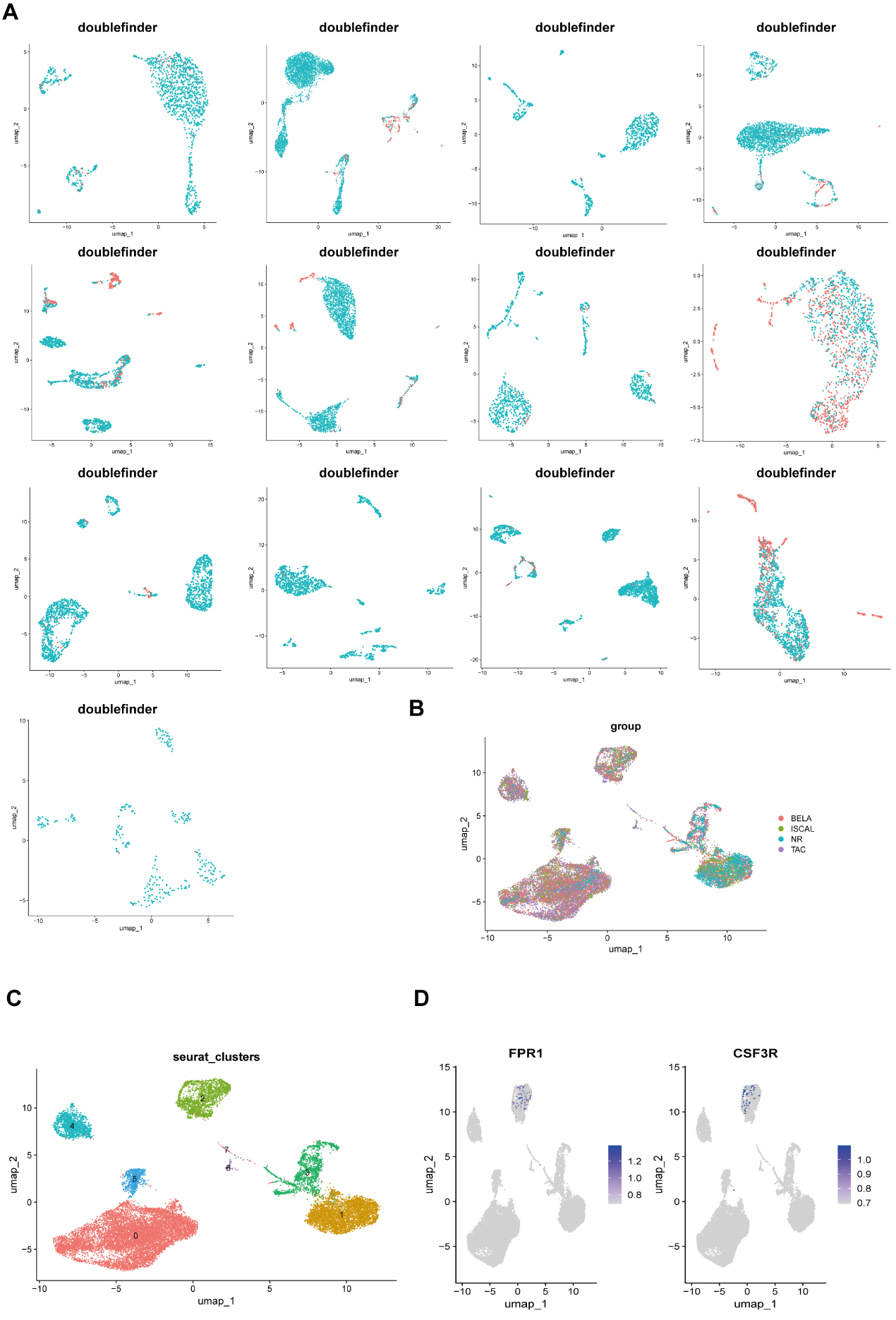


**Supplementary Figure** **4: Integration of Datasets.**

(A) Data deduplication. (B) Data de-batching. (C) Data clustering. (D) The expression levels of FPR1 and CSF3R in different cell types.


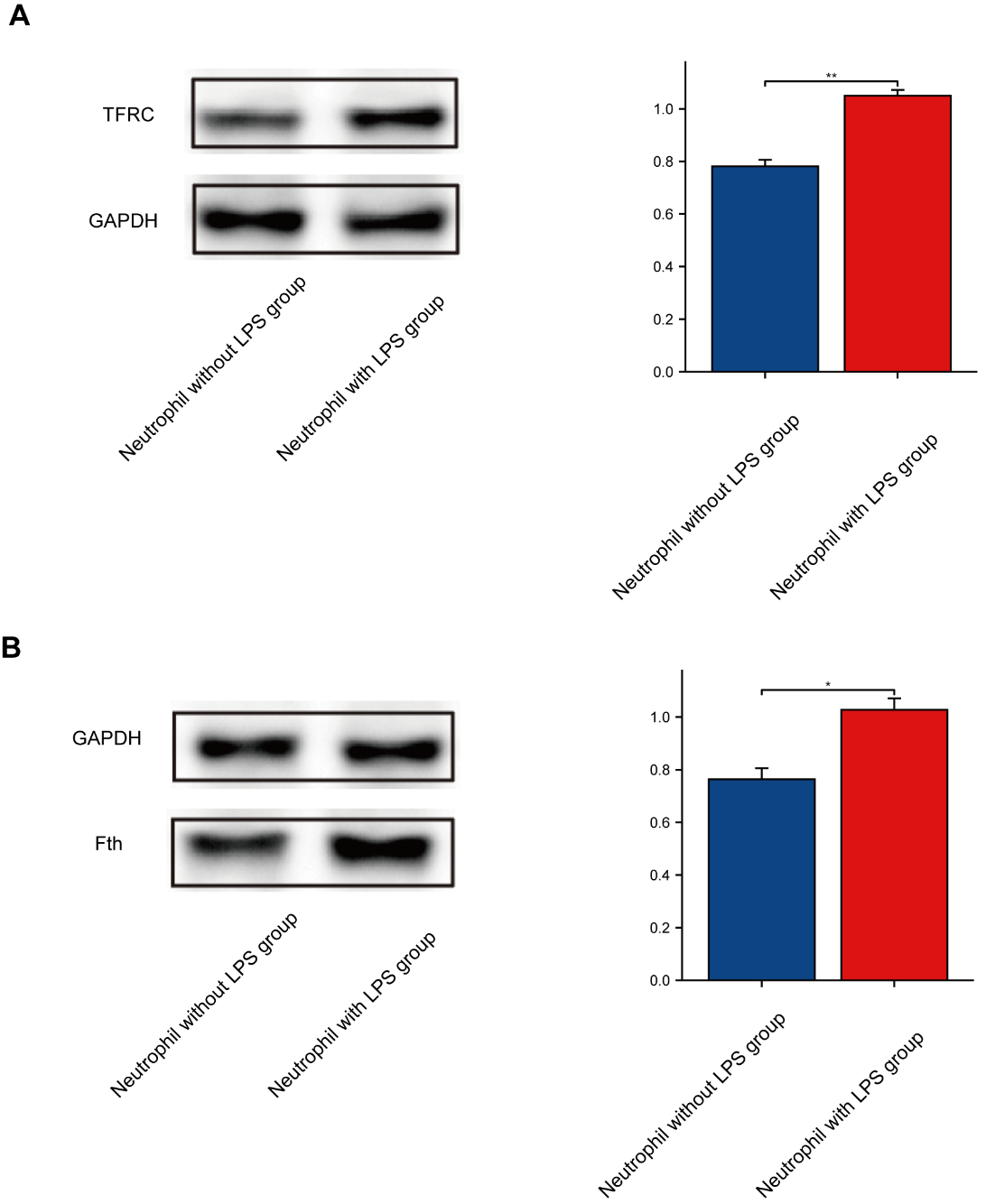


**Supplementary Figure** **5:** The protein expression levels of iron metabolism-related indicators in neutrophils without LPS stimulation and in neutrophils subjected to LPS stimulation. (A) The protein expression level of TFRC in neutrophils with/without LPS. (B) The protein expression level of Fth in neutrophils with/without LPS. n = 3, *, p < 0.05; **, p < 0.01.

**
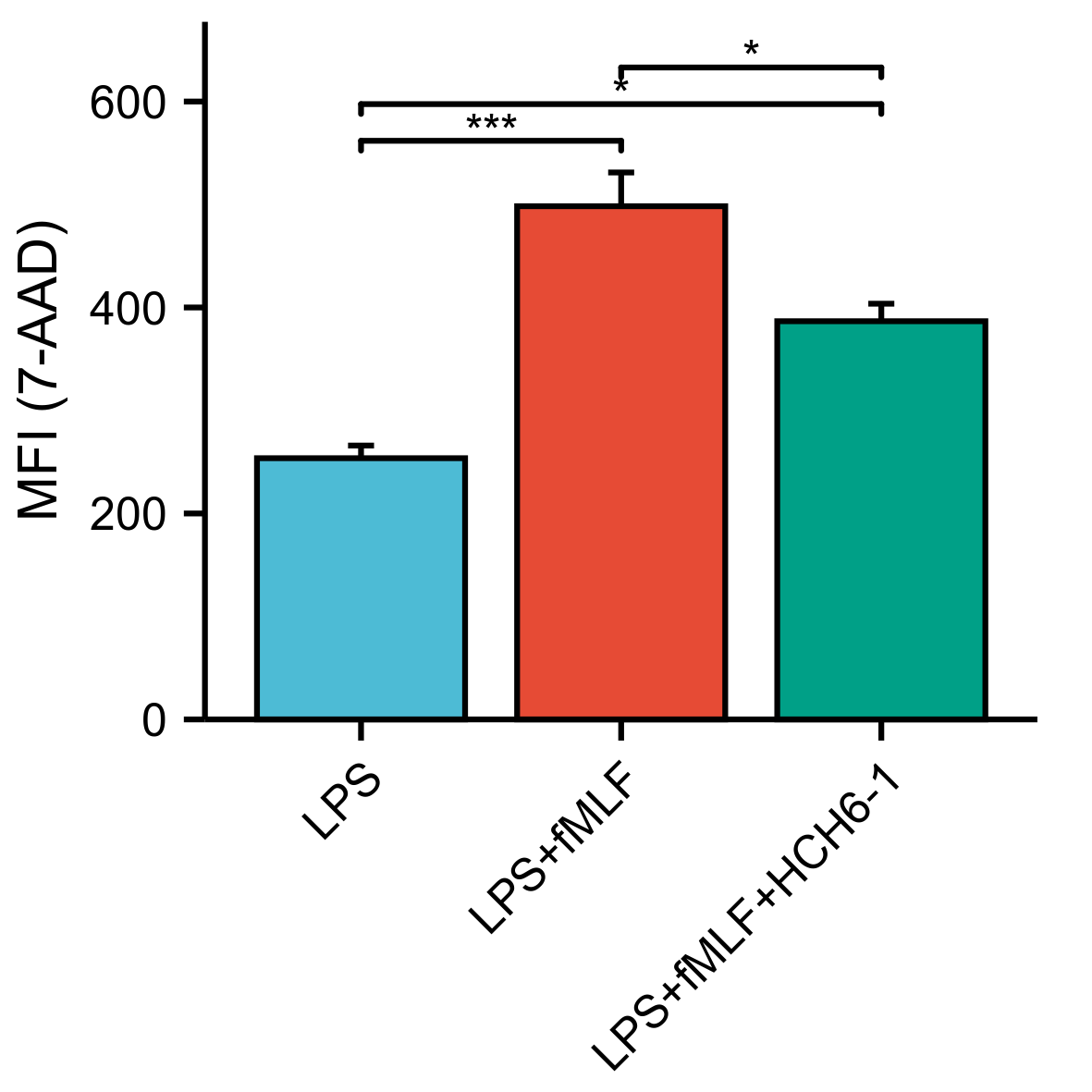
**

**Supplementary Figure** **6:** Mean Fluorescence Intensity of 7-AAD in neutrophils of each group. MFI: Mean Fluorescence Intensity, n = 3, *, p < 0.05; ***, p < 0.001.
