# Supplementary material for: FPR1 affects acute rejection in kidney transplantation by regulating iron metabolism in neutrophils
Source: Mol Med. 2025 Jan 23;31:23. doi: 10.1186/s10020-025-01077-w (PMC11758745; doi:10.1186/s10020-025-01077-w)
Supplement: Supplementary file 4 — Supplementary Material 4 [file 10020_2025_1077_MOESM4_ESM.docx]

| group | sample | GAPDH | TFRC |
| --- | --- | --- | --- |
| Control | sample1 | 19.49 | 24.05 |
| Control | sample2 | 19.47 | 24.05 |
| Control | sample3 | 19.44 | 23.94 |
| Control | sample4 | 19.44 | 24.05 |
| LPS | sample1 | 19.71 | 23.16 |
| LPS | sample2 | 19.83 | 23.25 |
| LPS | sample3 | 19.78 | 23.22 |
| LPS | sample4 | 19.89 | 23.43 |
| LPS+H6 | sample1 | 18.3 | 22.16 |
| LPS+H6 | sample2 | 18.27 | 22.18 |
| LPS+H6 | sample3 | 18.24 | 22.07 |
| LPS+H6 | sample4 | 18.25 | 22.04 |

**RT-PCR results for TFRC**
