# Supplementary material for: FPR1 affects acute rejection in kidney transplantation by regulating iron metabolism in neutrophils
Source: Mol Med. 2025 Jan 23;31:23. doi: 10.1186/s10020-025-01077-w (PMC11758745; doi:10.1186/s10020-025-01077-w)
Supplement: Supplementary file 5 — Supplementary Material 5 [file 10020_2025_1077_MOESM5_ESM.docx]

| **LPS** | **LPS+fMLF** | **LPS+fMLF+HCH6** |
| --- | --- | --- |
| **214** | **251** | **236** |
| **205** | **267** | **230** |
| **200** | **263** | **240** |

**ELISA results for NE (ng/mL)**

**ELISA results for TNF-α (pg/mL)**

| **LPS** | **LPS+FMLf** | **LPS+FMLf+HCH6-1** |
| --- | --- | --- |
| **1483** | **1934** | **1673** |
| **1461** | **1931** | **1667** |
| **1469** | **1925** | **1661** |
